# Trisomy 21 Alters DNA Methylation in Parent-of-Origin-Dependent and -Independent Manners

**DOI:** 10.1371/journal.pone.0154108

**Published:** 2016-04-21

**Authors:** Antônio Francisco Alves da Silva, Filipe Brum Machado, Érika Cristina Pavarino, Joice Matos Biselli-Périco, Bruna Lancia Zampieri, Ronaldo da Silva Francisco Junior, Pedro Thyago Mozer Rodrigues, Douglas Terra Machado, Cíntia Barros Santos-Rebouças, Maria Gomes Fernandes, Susana Marina Chuva de Sousa Lopes, Álvaro Fabricio Lopes Rios, Enrique Medina-Acosta

**Affiliations:** 1 Laboratory of Biotechnology, Universidade Estadual do Norte Fluminense Darcy Ribeiro, Campos do Goytacazes, Rio de Janeiro, Brazil; 2 Molecular Identification and Diagnostics Unit, Hospital Escola Álvaro Alvim, Campos dos Goytacazes, Rio de Janeiro, Brazil; 3 Graduate Program in Biosciences and Biotechnology, Center for Biosciences and Biotechnology, Universidade Estadual do Norte Fluminense Darcy Ribeiro, Rio de Janeiro, Brazil; 4 Postgraduate Program in Biosciences and Biotechnology, Center for Biosciences and Biotechnology, Universidade Estadual do Norte Fluminense Darcy Ribeiro, Rio de Janeiro, Brazil; 5 Faculdade de Medicina de São José do Rio Preto, São Paulo, Brazil; 6 Department of Genetics, Universidade do Estado do Rio de Janeiro, Rio de Janeiro, Rio de Janeiro, Brazil; 7 Department of Anatomy and Embryology, Leiden University Medical Center, Leiden, South Holland, The Netherlands; University of Bonn, Institute of Experimental Hematology and Transfusion Medicine, GERMANY

## Abstract

The supernumerary chromosome 21 in Down syndrome differentially affects the methylation statuses at CpG dinucleotide sites and creates genome-wide transcriptional dysregulation of parental alleles, ultimately causing diverse pathologies. At present, it is unknown whether those effects are dependent or independent of the parental origin of the nondisjoined chromosome 21. Linkage analysis is a standard method for the determination of the parental origin of this aneuploidy, although it is inadequate in cases with deficiency of samples from the progenitors. Here, we assessed the reliability of the epigenetic 5^m^CpG imprints resulting in the maternally (oocyte)-derived allele methylation at a differentially methylated region (DMR) of the candidate imprinted *WRB* gene for asserting the parental origin of chromosome 21. We developed a methylation-sensitive restriction enzyme-specific PCR assay, based on the *WRB* DMR, across single nucleotide polymorphisms (SNPs) to examine the methylation statuses in the parental alleles. In genomic DNA from blood cells of either disomic or trisomic subjects, the maternal alleles were consistently methylated, while the paternal alleles were unmethylated. However, the supernumerary chromosome 21 did alter the methylation patterns at the *RUNX1* (chromosome 21) and *TMEM131* (chromosome 2) CpG sites in a parent-of-origin-independent manner. To evaluate the 5^m^CpG imprints, we conducted a computational comparative epigenomic analysis of transcriptome RNA sequencing (RNA-Seq) and histone modification expression patterns. We found allele fractions consistent with the transcriptional biallelic expression of *WRB* and ten neighboring genes, despite the similarities in the confluence of both a 17-histone modification activation backbone module and a 5-histone modification repressive module between the *WRB* DMR and the DMRs of six imprinted genes. We concluded that the maternally inherited 5^m^CpG imprints at the *WRB* DMR are uncoupled from the parental allele expression of *WRB* and ten neighboring genes in several tissues and that trisomy 21 alters DNA methylation in parent-of-origin-dependent and -independent manners.

## Introduction

Trisomy 21 (Down syndrome) is the most common autosomal aneuploidy that is compatible with life (average rate of 1/400-800 live births; average life expectancy of 55 years) [1]. The supernumerary chromosome 21 results from meiotic nondisjunction errors in approximately 90–95% of cases during oogenesis [2, 3]. Thus, most individuals with Down syndrome inherit two maternal complete and free copies of chromosome 21. Advanced maternal age increases the risk of pregnancy with trisomy 21 [4], while the evidence for an association with paternal age is inconsistent [5–8]. Therefore, the extremely skewed disparity observed between the maternal and paternal meiotic errors at the origin of chromosome 21 nondisjunction is mainly explained by the effect of advanced maternal age.

Although individuals with Down syndrome share phenotypically distinctive traits including clinical manifestations of atypical and segmental accelerated aging [9], the syndrome exhibits a large variety of physical stigmata that are unevenly represented among probands. Some of the defects may constitute severe pathologies (i.e., cognitive dysfunction, acute lymphoblastic leukemia, congenital heart disease, premature aging and Alzheimer disease-like neuropathology). Despite the considerable number of clinical studies on the intellectual and physical disabilities associated with trisomy 21, and owing to the small proportion of paternally inherited cases, there is still no conclusive information regarding whether parent-of-origin (maternal *versus* paternal) genetic factors contribute differentially to the observed phenotypic variation. For example, in one study [10], no significant difference was found in the distribution of phenotypic clinical findings between Down syndrome patients with maternal (n = 150) and paternal (n = 8) origin of the nondisjunction errors. In another study [11], congenital heart defects, high arched palate, and short fingers occurred less frequently in cases with a paternally-derived extra chromosome 21 (n = 8) than in cases with a maternally-derived extra chromosome 21 (n = 28). The main caveats in those reports are the use of a limited number of polymorphic markers in the first study mentioned and the use of nucleolar organizer region heteromorphisms in the second study mentioned for the conclusive determination of the paternal origin of the nondisjunction errors.

Considering the scenario of genomic imprinting on chromosome 21 [12], the parental origin of the supernumerary chromosome 21 will contribute differentially to the development of some defects in a parent-dependent manner in Down syndrome. Notably, the uncommon condition of a normal phenotype with maternal [13–15] or paternal [16, 17] uniparental disomy (UPD) of chromosome 21 does not rule out the possibility of genomic imprinting of non-essential genes. In fact, UPD involving chromosomes containing imprinted genes does not necessarily reveal an imprinted pathological disorder with clinical consequences [18]. Importantly, the extra copy of chromosome 21 causes a genome-wide [19, 20] domain pattern [20] of dysregulation of gene expression, and it affects the DNA methylation levels differentially at distinct CpG dinucleotide sites [21, 22]. At present, it is also is unknown whether those effects are dependent or independent of the parental origin of the nondisjoined chromosome 21.

The parental origin of the nondisjoined chromosome 21 can be established experimentally in nuclear trios (mother, father, and proband) by linkage analysis using highly polymorphic DNA markers (i.e., genotyping with short tandem repeats, STRs) [23]. Such expert analysis is difficult when DNA samples from the progenitors are unavailable. Here, we assessed the dependability of reversible epigenetic molecular imprints resulting in germline-specific 5^m^CpG for the discrimination of the parental origin of chromosome 21 nondisjunction. For that purpose, we developed a PCR assay based on maternally derived allele methylation at a differentially methylated region (DMR) located in the tryptophan-rich basic protein *WRB* gene. Two research groups recently identified the target maternal-of-origin 5^m^CpG imprints at the *WRB* DMR using genome-wide methylation chip technology [24, 25]. The occurrence of a DMR in the *WRB* gene warranted campaigning it as the first candidate maternally imprinted gene (i.e., paternally expressed) on the human chromosome 21. In contrast to the uniparental inheritance pattern of allele expression determined by imprinting, one study reported alternate (i.e., opposing) monoallelic expression of the maternal or the paternal *WRB* alleles in different human fetal tissues from the same embryo [25], a pattern consistent with random monoallelic expression, rather than with imprinting. Thus, we also reappraised the candidate imprinting status of the *WRB* gene. We performed single-nucleotide polymorphism primer extension (SNuPE) at informative 3´-UTR SNP variants in DNA from blood cells, and in human embryonic stem cell lines (hESCs). Furthermore, we conducted an integrative and comparative epigenomic computational analysis using transcriptome RNA sequencing (RNA-Seq) public repositories, essentially adopting the frameworks recently described to explore enrichment-based sequencing data [26, 27].

Finally, we also evaluated the impact of the parental origin of the supernumerary chromosome 21 on the methylation statuses at CpG sites in the *RUNX1* (chromosome 21) and *TMEM131* (chromosome 2) genes, the methylation levels of which are altered with trisomy 21 [21, 22, 28].

## Materials and Methods

### Ethics Statement

Peripheral blood samples from participating control and Down syndrome nuclear families were collected with written informed consent. For infants and children with Down syndrome, a surrogate consent procedure was used, whereby the next of kin or a legally authorized representative consented in writing on the behalf of the participants. The subjects were included from projects approved by the Ethics Committee of the Faculdade de Medicina de Campos, Brazil (approval code FR-278769), the Faculdade de Medicina de São José do Rio Preto, Brazil (HCRP 5810/2009) and the Universidade do Estado do Rio de Janeiro, Brazil (040/2005). The main objectives of those projects were to develop molecular genetic tests for the determination of the parental origin of the nondisjunction of human chromosome 21 and to screen for parent-of-origin genetic risk factors for trisomy 21. This study was conducted according to the principles expressed in the Declaration of Helsinki.

### Subjects

We included 70 nuclear families (mother, father, and child) of either male or female index cases with the full free trisomy 21 status established by conventional karyotyping (G band) in at least 20 cells. We also included 20 families of children with no trisomy (controls). The average maternal age of index cases was 31.6 years (ranging from 16 to 44 years). After determining the parental origin of the nondisjunction error for at least three informative STRs and selecting for the informative SNPs in the target *loci*, 15 trios with a maternal origin (MT21), six with a paternal origin (PT21) and 13 control trios (N21) met the criteria for the investigation of parental-of-origin allele methylation and allele expression.

### Cell Lines

Cell pellets from the commercial, established human embryonic stem cell lines HUES 1, HUES 3, HUES 5, HUES 7, and HUES 15 were kindly provided by Christine L. Mummery, from the Department of Anatomy and Embryology, Leiden University Medical Center, Leiden, The Netherlands. General information regarding these cell lines is available at the NIH Human Embryonic Stem Cell Registry (http://grants.nih.gov/stem_cells/registry/current.htm)

### DNA and RNA Extraction

Human genomic DNA and total RNA from freshly drawn peripheral blood samples were extracted using phenol-chloroform and ethanol precipitation [29] and TRIzol reagent (Invitrogen, Carlsbad, CA, USA), respectively, as previously described [30].

### Determination of the Parental Origin of Chromosome 21 Nondisjunction through Linkage Analysis

To select cases of trisomy 21 of either maternal or paternal origin of the nondisjunction error, we performed linkage analysis by quantitative fluorescence PCR genotyping nuclear trios at highly polymorphic STRs using fluorochrome-labeled primers and separating the amplimers by high-resolution capillary electrophoresis. The physical locations and primer sequences are shown in S1 Table. We determined the parental origin of the extra copy of chromosome 21 by comparing the genotype of the index case with the parental genotypes at the informative STR *loci*. We identified the parental alleles according to the following possible scenarios of allele segregation: index case presenting with a triallelic pattern exhibiting allele ratios of approximately 1:1:1 or a biallelic pattern exhibiting a consistent allele ratio of 2:1. The observed genotypes are shown in S2 Table.

### Assessment of Heritable Epigenetic Methylation Imprints

To infer the methylation status at a given CpG island (CGI), we developed CGI-specific methylation-sensitive restriction enzyme-based PCR triplex assays (MSRE-PCR). In one tube reaction, each assay amplifies the target CGI and two other genomic regions as a control for the efficiency of the restriction enzyme digestion and to normalize the estimate ratios of the restriction enzyme-resistant 5^m^CpG sites. Allele-specific methylation was determined by interrogating informative SNPs neighboring the target CGIs. Information regarding the physical location of the selected target CGIs, 5^m^CpG-sensitive restriction enzymes, SNPs and primer sequences used in the assays is included in S1 Table. Because of the potential diagnostic value of the *WRB* DMR (CGI-2) for the discrimination of the parental origin of nondisjunction of the supernumerary chromosome 21, we describe its specific MSRE-PCR assay here. On untreated gDNA, the assay generates three FAM-labeled amplimers of different lengths. On *Hha*I treated gDNA, the combined pattern of the amplimers is used to determine one of three possible statuses of methylation: hypomethylated, hemimethylated or hypermethylated. The genomic amplimers are: (1) The *WRB* DMR target region (observed amplicon size: 254 bp), encompassing the recognition sites for *Hha*I, and the rs2299739 and rs2244352 SNPs (S1 Table). (2) The known unmethylated *ESCO2* core promoter CGI (observed amplicon size ranging 248 to 250 bp), encompassing many *Hha*I recognition sites. The *ESCO2* core promoter CGI was originally featured as a query target in the SALSA MS-MLPA ME030 probemix commercial kit (MRC Holland). This genomic amplimer provides information regarding the efficiency of the restriction enzyme reaction. (3) A region (observed amplicon size ranging 269 to 271 bp) genetically linked to the *WRB* DMR but lacking *Hha*I recognition sites. This amplimer serves as a normalization reference. Parental allele methylation statuses at the *WRB* DMR were assessed by interrogating neighboring SNPs using a non-fluorescent uniplex version of the MSRE-PCR. Next, we carried out SNuPE assays to genotype the methylated alleles, refractory to digestion with the restriction enzyme, using SNaPshot technology (Thermo Fisher Scientific, Waltham, MA, USA). To validate the parental allele-specific methylation statuses, we used the *McrBC* restriction endonuclease that acts upon DNA containing methylcytosine on one or both strands [31]. We used the same experimental design to profile the methylation statuses at 5^m^CpG-restriction enzyme sensitive sites located at the *WRB* CGI-1 and CGI-3. To evaluate the impact of the parental origin of the extra copy of chromosome 21 in the methylation of CpG sites at the *RUNX1* and *TMEM131* genes, we developed gene-specific methylation-sensitive restriction enzyme MSRE-PCR assays (S1 Table).

We calculated the ratio of restriction enzyme-resistant 5^m^CpG sites at the queried CGIs using the following equation:

$$\%\text{Meth}\;=\left\{\frac{\left[\frac{C-(C\;\times \;f_{AB})}{C-(C\;\times (f_{AB})+D+(D\;\times \;f_{AB})}\right]}{\left[\frac{D+(D\;\times \;f_{AB})}{C-(C\;\times \;f_{AB})+D+(D\;\times \;f_{AB})}\right]}\right\}\times 100,\;f_{AB}=\left[\left(\frac{\text{A}\times 100}{\text{A}\;+\;\text{B}}\right)-50\right]\;\times 0.02$$

where, ***C*** is the value of the area under the peak (AUP) of the queried CGI amplimer from the restriction enzyme digested DNA sample; ***D*** is the AUP of the negative amplimer from the restriction enzyme digested DNA sample, and ***f***_***AB***_ is the correction factor that accounts for the lack of symmetry and imbalance observed between the AUP for the queried GCI amplimer (**A**) and the negative amplimer (**B**) obtained from the undigested DNA sample.

### An Androgenetic Reference Sample of Methylation Statuses in Male Germline Derivatives

The homogenous androgenetic nature of the hydatidiform mole was determined by comparing the genotypes (S3 Table) of the two samples of the same specimen with that of a peripheral blood sample from the donor by quantitative fluorescence PCR using the AmpF*l*STR Identifiler system (Thermo Fisher Scientific, Waltham, MA, USA).

### Assessment of Transcriptional Allele Expression

To discriminate between the possible biallelic and the monoallelic patterns of transcriptional expression of the *WRB* gene, we interrogated 3´-UTR SNPs in cDNAs obtained from heterozygous blood donors and hESCs using SNaPshot technology. For comparison, we genotyped SNPs at two paternally imprinted reference genes: *H19* [32] and *ATP10A* [33]. Primer sequences are shown in S1 Table.

### High-Resolution Capillary Electrophoresis for Separation of Fluorochrome-Labeled Amplimers

Amplimers were analyzed on an automated laser fluorescent ABI PRISM 310 Genetic Analyzer (Thermo Fisher Scientific, Waltham, MA, USA). The electropherograms were generated using the dedicated GeneScan® Analysis and Genotyper® software version 3.7 packages and GeneMapper® ID version 3.2 (Applied Biosystems ® from Thermo Fisher Scientific, Waltham, MA, USA).

### The Methylation Statuses at the *WRB* CpG Islands in Methylome Public Datasets

To examine the methylation patterns at the CGI mapped to the reference *WRB locus*, we performed an integrative and comparative epigenomic computational analysis by viewing the Smith Lab Public DNA methylation track hub [34] and the 111 distinct epigenomes from the Roadmap Consortium [35] at the UCSC Genome Browser [36, 37]. The Smith Lab Public DNA methylation track hub comprises a pre-loaded set of 183 analyzed human methylomes from bisulfite sequencing experiments from brain [38–40], hESCs [41], induced pluripotent stem cells (iPSCs) [42] and blood cells [43–45]. The rates of 5^m^CpG along the chromosome 21 in human oocytes [46] were displayed as a custom track in the UCSC Genome Browser. We graphically displayed the predicted promoters and CTCF and POL2 binding sites at the UCSC interface using the Ensembl Regulatory Build and Transcription Factor Binding track resource [47].

### Identification of DNA Motifs Mapping at the *WRB* DMR

To search for *cis*-acting motifs that may be implicated in the differential epigenetic statuses of the *WRB* DMR, we queried published libraries of DNA-binding proteins with the FASTA reference sequence for the *WRB* DMR using online search DNA motif programs [48, 49]. We also used Tandem Repeat Finder [50] to scan the *WRB* DMR DNA reference sequence for the presence of ungapped (regular, fixed-length patterns) DNA repeat elements. The downstream analysis for the identified array is based on the rationale that *cis* elements constitute platforms on which the interactions with site-specific DNA-binding factors are built to establish and maintain epigenomic modifications [51]. To investigate whether the identified DNA motifs correspond to transcription factor binding sites, we compared the DNA motifs against a database of known motifs using the TOMTOM tool [52], which ranks each suitable match to the query and displays motif web logos. To address whether the motifs found in the *WRB* DMR are present in other *loci*, we searched the entire reference genome sequence using the FIMO tool [53] from the MEME program suite [48] available online at http://meme-suite.org/. The conservation of the array of DNA motifs was investigated by lifting over the coordinates in the reference genomes of vertebrates using the *LiftOver* tool from the UCSC Genome Browser [36] and submitting the lifted FASTA sequences to the motifs analysis. The coordinates of the tandem repeat motif array were then graphically displayed in the UCSC Genome Browser [36, 37] using the custom track tool.

### Combinatorial Histone Modification Expression Signatures

We compared the common combinatorial histone modification expression patterns across the DMRs in 29 known maternally and two paternally imprinted genes [46] with that in the candidate imprinted *WRB* gene by displaying the sequence features and the activating or repressive histone marks in the UCSC genome browser using annotation and track hubs. As testable predictions of the epigenetic status, we used the following two modification modules defined by Wang and collaborators in human CD4+ T cells [54]. The 17-histone modification activation backbone module: H2A.Z, H2BK5ac, H2BK12ac, H2BK20ac, H2BK120ac, H3K4ac, H3K4me1, H3K4me2, H3K4me3, H3K9ac, H3K9me1, H3K18ac, H3K27ac, H3K36ac, H4K5ac, H4K8ac, and H4K91ac. The 5-histone modification repressive module: H3K27me3, H3K27me2, H3K9me3, H3K9me2, and H4K20me3.

### Reappraisal of the Candidate Maternally Imprinting Status of the *WRB* Gene Using Secondary Analysis of Massive Parallel RNA Sequences

We measured allelic imbalance at likely heterozygous *loci* that map within a 4-Mb chromosomal region centered at the *WRB* gene by querying RNA sequence read archive (SRA) public data repositories. We included exon, 5´-UTR, 3´-UTR and ncRNA SNPs being 159 SNPs with MAF > 0.1 (i.e., expected global heterozygosity rate of 0.18) as testable predictions and 4 SNPs with MAF <0.05 as a control for monoallelic expression (S4 Table), mapping to within 35 genes and ncRNAs. As a reference, we used SNPs at the *SNURF* and *H19* genes (S1 Table), which are both imprinted in every human tissue tested so far [55]. The public databases are available at NCBI Gene Expression Omnibus GEO (http://www.ncbi.nlm.nih.gov/geo/) [56], EMBL-EBI ArrayExpress (https://www.ebi.ac.uk/arrayexpress/) [57] and the Human Protein Atlas (http://www.proteinatlas.org/). We used the SRA nucleotide search expression online tool from the NCBI Browser (http://trace.ncbi.nlm.nih.gov/Traces/sra/) to download FASTQ filtered reads. The SRA Nucleotide Search Expression tool returns biological reads for an RNA-Seq spot containing a sequence substring encompassing a given SNP. Each sequence substring was limited to 29 characters in 4NA alphabet including the International Union of Pure and Applied Chemistry (IUPAC) substitution codes for the query SNPs (S1 and S4 Tables). We used IUPAC genomic reference to correct for reference allele preference during alignment [58]. We restricted the analysis to two series of experiments. The first included 1,012 human RNA-Seq sample runs (S5 Table) selected from the 4,978 unsorted accessions recently analyzed by Deelen et al. (2015) [59]. The second included 212 SRA accessions from public repositories and sorted into 15 primary tissue sources (S6 Table) by their reported Biosample and Bioproject unicity (i.e., one sample, not a mixture, from a single donor). We quantified the allele fractions only in runs that yielded a depth of at least 80 reads, with a quality of base calls > Q30, increasing the variant confidence detection to a probability of correct SNP call of 0.999 provided a read depth (coverage) of 40 for a theoretical heterozygous position [60]. We categorized the patterns of allele expression as monoallelic, biallelic or biallelic imbalance. The criteria were: monoallelic if the allele fractions were < 0.15 or > 0.85; biallelic if the allele fractions ranged from 0.35 to 0.65; and biallelic imbalance if the allele fractions ranged from > 0.15 to <0.35 or > 0.65 to <0.85. We used a chi-square test to evaluate whether the allele-specific read counts deviated from the expected proportions (50/50) [58]. To map FASTQ formatted, filtered spot RNA raw sequence data to the hg19 reference sequence we used a flexible workflow created with the web-based Galaxy tool suite (https://usegalaxy.org/) [61]. The workflow incorporates the use of the following software tools: FastQC (read quality control check) [62], FASTQ Groomer, Filter by quality (Phred score quality cut-off: 25; minimum percentage: 90), removal of sequencing artifacts, Bowtie2, BWA, BAM-to-SAM, Filter SAM or BAM, output SAM or BAM (reads with maximum 01 variant or reads with no variant), and FreeBayes (Bayesian genetic variant detector). We visualized the filtered and aligned reads using the UCSC graphical interface.

## Results

### The Methylation Statuses at the *WRB* CpG Islands

**Fig 1:**
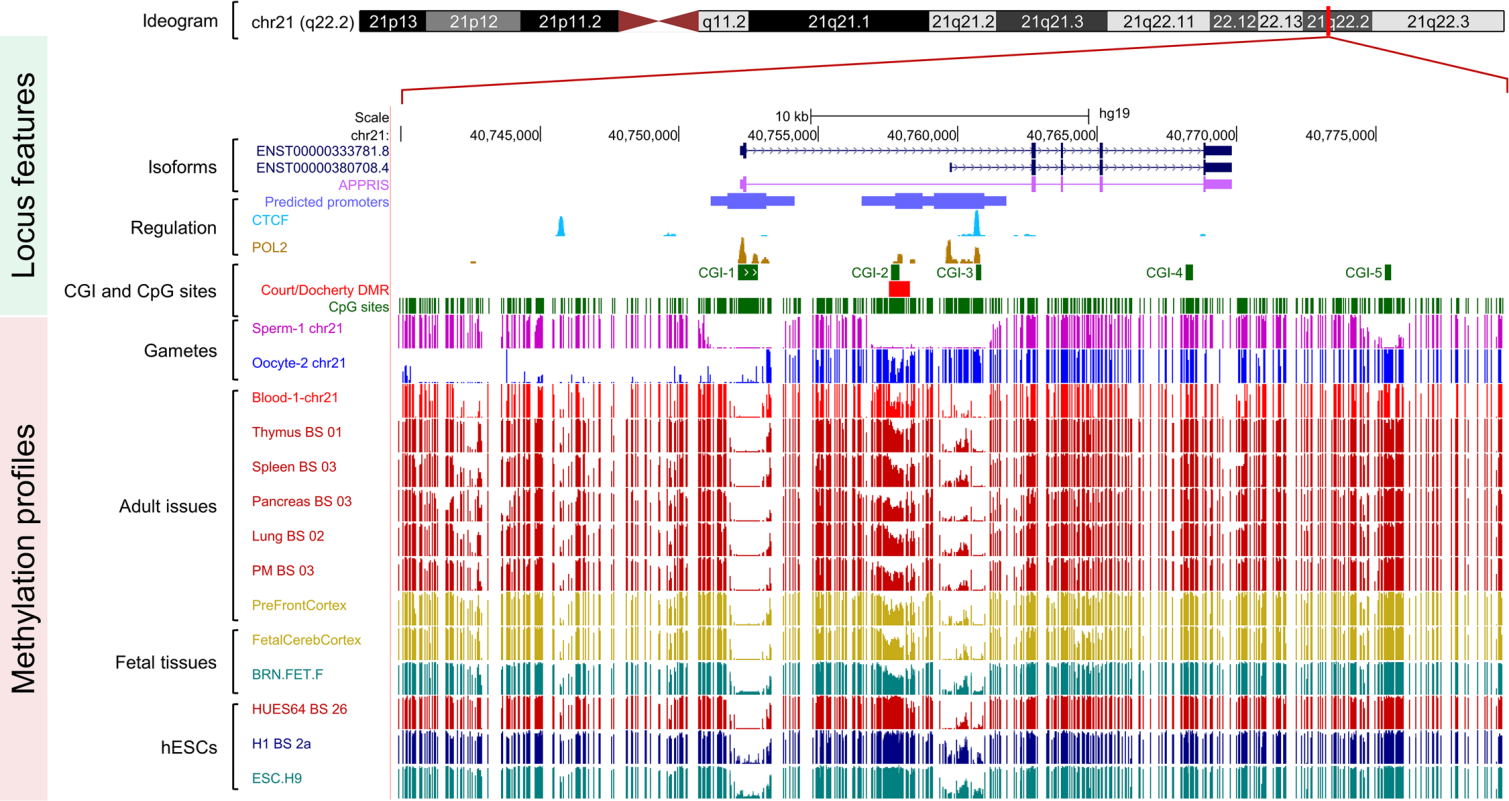
The methylation statuses at the CpG islands of the *WRB* gene in methylome public datasets. Chromosomal, physical map positions and sequence features of the reference *WRB* gene *locus* showing the methylation profiles across the region, with an emphasis on the five annotated CpG islands (CGI-1 to CGI-5). The features depicted are from custom and public tracks for (from top to bottom) the exon organization of the principal (ENST00000333781.8) and alternative (ENST00000380708.4) splice *WRB* isoforms (variants 1 and 2 in dark blue) (UCSC Genes [36]), the species-conserved principal transcript (ENST00000333781 in pink) (APPRIS [75]), the Ensembl Regulatory Build CTCF and POL2 activity and predicted promoters [76], CpG islands, CpG sites, and regulation and methylome studies indicated in the Materials and Methods section. The CGI-2 is located in the differentially methylated region (DMR) reported by Court and collaborators [24] and Docherty and collaborators [25] (depicted in red in the custom track named Court/Docherty DMR), from which *WRB* was classified originally as a novel candidate, maternally imprinted gene (i.e., paternal-origin allele expressed). The custom tracks containing the sperm and oocyte DNA methylation signals correspond to the supplementary data reported by Okae *et al*. [46]. Screenshot generated using the UCSC Genome Browser hg19 (http://genome.ucsc.edu).

The *WRB* reference sequence locus contains five annotated CpG islands, CGI-1, CGI-2, CGI-3, CGI-4, and CGI-5 comprising 64, 27, 19, 17 and 18 CpG dinucleotide sites, respectively (Fig 1). CGI-1 encompasses the 5´-UTR and exon 1 of the ENST00000333781.8 reference *WRB* transcript variant 1 (long variant), and maps to a predicted promoter region. In public methylome databases, CGI-1 is essentially unmethylated in gametes and in somatic and embryonic cells and tissues (Fig 1). CGI-2 maps to a second predicted promoter region, located upstream of the TSS of the ENST00000380708.4 reference *WRB* transcript variant 2 (short variant) (Fig 1). CGI-2 is located within the differentially methylated region (DMR) reported by Court and collaborators [24] and Docherty and collaborators [25] for which *WRB* was classified as a candidate maternally imprinted gene (i.e., paternal-origin allele expressed). CGI-2 is differentially methylated in oocytes *versus* sperm [46], partially methylated in adult somatic tissues, and hypermethylated in embryonic cells and tissues (Fig 1). CGI-3 is differentially methylated in female and male gametes, essentially unmethylated in adult cells and tissues, and hypomethylated in embryonic stem cells (Fig 1). CGI-4 is hypermethylated in gametes, in all somatic cells, and in tissues (Fig 1). CGI-5 is differentially methylated in the gametes and is ubiquitously hypermethylated in somatic cells and tissues (Fig 1).

**Fig 2:**
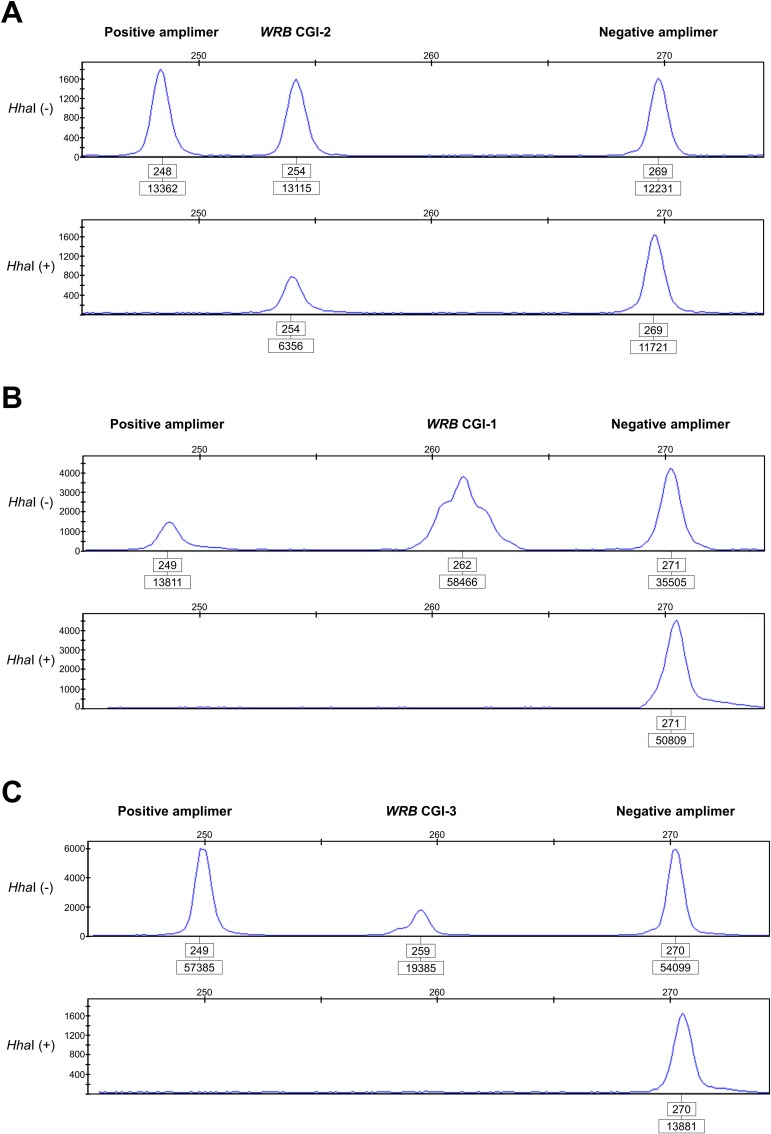
Experimental validation of the 5^m^CpG statuses at the *WRB* CGIs. (**A**) A consistent hemimethylated pattern at the *WRB* CGI-2 revealed in a representative control disomic DNA sample (blood) using the Hha*I* methylation-sensitive restriction enzyme-based PCR triplex assay developed in this study. Electropherograms of the amplimers generated from either undigested genomic DNA (upper panel) or DNA digested with *Hha*I (lower panel) genotyped via quantitative fluorescent PCR. The positive amplimer refers to a *locus* in the *ESCO2* gene with constitutively hypomethylated CpG dinucleotides at the target restriction enzyme sites (100% susceptible to *Hha*I digestion). The negative amplimer refers to a *WRB* region that lacks *Hha*I sites, and is, therefore, refractory to enzymatic digestion. The numbers in the upper boxes correspond to the amplimer lengths in base pairs while those in the lower boxes refer to the areas under the peak of the amplimer. In this representative DNA sample, the ratio of 5^m^CpG sites at the *WRB* CGI-2 was 50.6%. In contrast, the assay revealed a consistent unmethylated pattern of CpG sites at the *WRB* CGI-1 (**B**) and *WRB* CGI-3 (**C**).

Using target-specific MSRE-PCR assays, we experimentally replicated the above methylation statuses at the *WRB* CGI-2 (Fig 2A), CGI-1 (Fig 2B), and CGI-3 (Fig 2C) in genomic DNA from disomic individuals. The overall average ratio of restriction enzyme-resistant 5^m^CpG sites at the *WRB* CGI-2 was 47.4% in disomic blood cells from adult donors (S7 Table). In hESCs, the overall average ratio of 5^m^CpG sites was 82.1% (S7 Table). In contrast, the assay revealed a consistent unmethylated pattern of CpG sites at the *WRB* CGI-1 (Fig 2B) and CGI-3 (Fig 2C).

**Fig 3:**
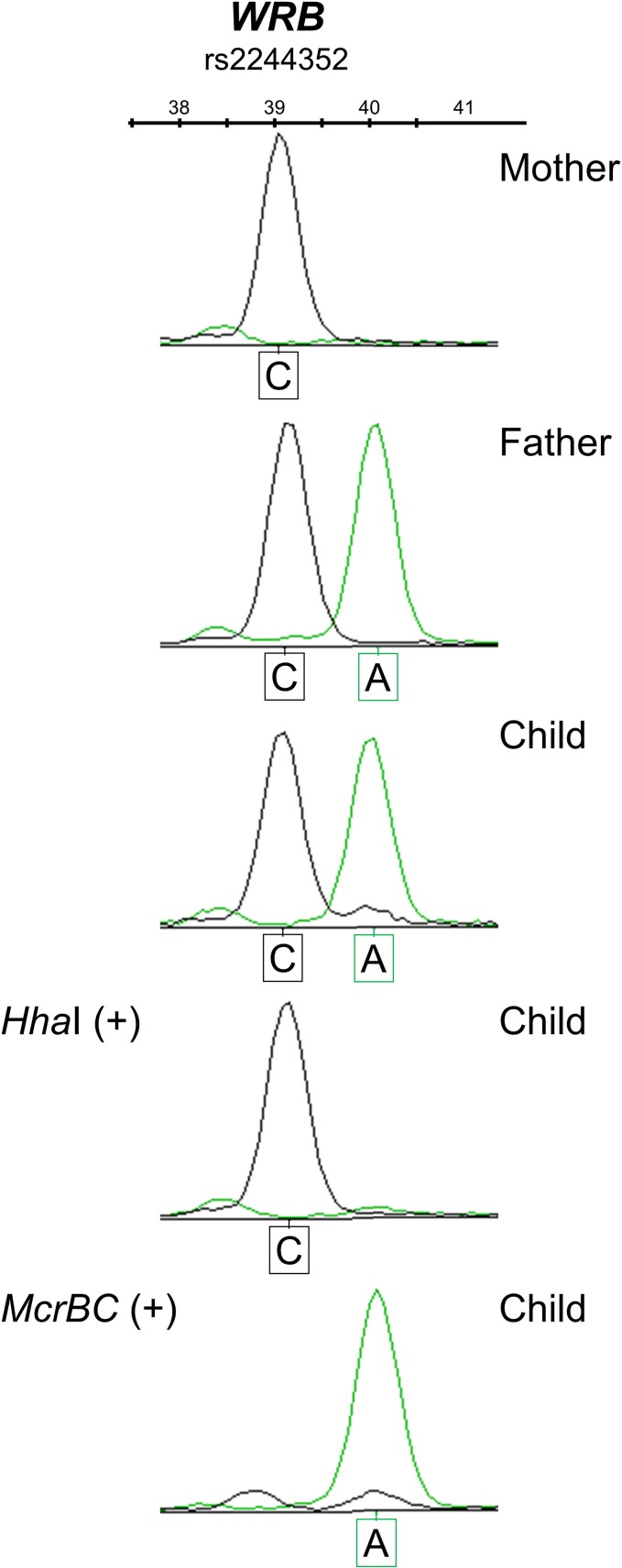
Maternal-of-origin-dependent imprinted methylation marks at the *WRB* CGI-2 DMR. 5^m^CpG-sensitive restriction endonuclease sites at the *WRB* CGI-2 DMR are differentially methylated on the maternal allele *versus* the paternal allele in a manner consistent with imprinting. Electropherograms of the genotype profiles in a control nuclear family, informative for the rs2244352 (C>A) SNP neighboring the *WRB* CGI-2 DMR. In the child, the maternal-derived C allele is 100% resistant to *Hha*I digestion (fully methylated), whereas the paternal-derived A allele is 100% susceptible to *Hha*I digestion (i.e., unmethylated). The parental allele-specific methylation statuses were validated using the *McrBC* restriction endonuclease that cleaves methylated DNA and, therefore, the unmethylated paternal allele, but not the maternally methylated allele, remains undigested.

The allele-specific methylation at the *WRB* CGI-2 DMR was assessed experimentally across the neighboring rs2244352 SNP in genomic DNA from peripheral blood cells of disomic heterozygous subjects. The maternal alleles were consistently methylated (i.e., refractory to digestion with *Hha*I and susceptible to digestion with *McrBC*) while the paternal alleles were unmethylated (i.e., susceptible to digestion with *Hha*I and refractory to digestion with *McrBC*) (Fig 3). Thus, the underlying genetic basis for the hemimethylated profiles observed at the *WRB* CGI-2 DMR is unequivocally due to the inheritance of specific maternal-allele 5^m^CpG imprints.

### Discrimination of the Parental Origin of Chromosome 21 Nondisjunction Using Heritable 5mCpG Imprints at the WRB CGI-2 DMR

We assessed the dependability of the known heritable epigenetic methylation [24, 25] resulting in maternal allele methylation imprints at the *WRB* CGI-2 DMR to ascertain the parental origin of chromosome 21 nondisjunctional events in trisomy 21 probands. For these experiments, the parental origin of the nondisjunctional events was unambiguously established by linkage analysis in nuclear families using highly polymorphic STRs. The genotypes and the segregating alleles are shown in S2 Table.

**Fig 4:**
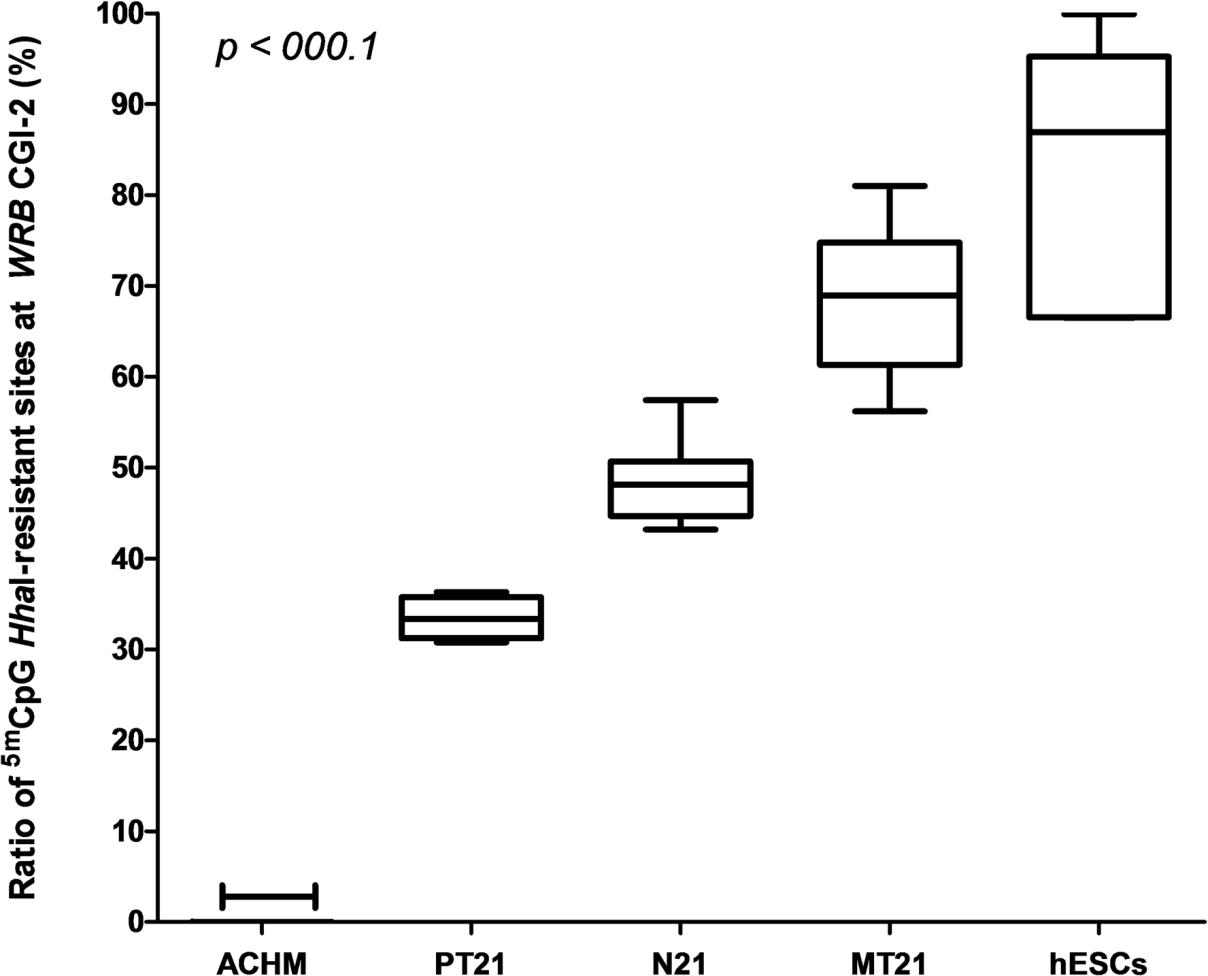
The maternal (oocyte)-derived allele methylation at the *WRB* CGI-2 distinguishes the parental origin of chromosome 21 nondisjunction events in Down syndrome probands. The proportion of *Hha*I-resistant 5^m^CpG sites at the *WRB* CGI-2 DMR in trisomic samples with a maternal origin (MT21) of the nondisjoined chromosome 21 is consistently and statistically higher than in trisomic samples with a paternally (PT21) derived extra copy of chromosome 21. In genomic DNA from an androgenetic hydatidiform mole (ACHM), the *WRB* CGI-2 is unmethylated, whereas it is hypermethylated in hESCs. In control disomic samples (N21) the *locus* is partially methylated, consistent with a hemimethylated state. All differences were statistically significant.

**Fig 5:**
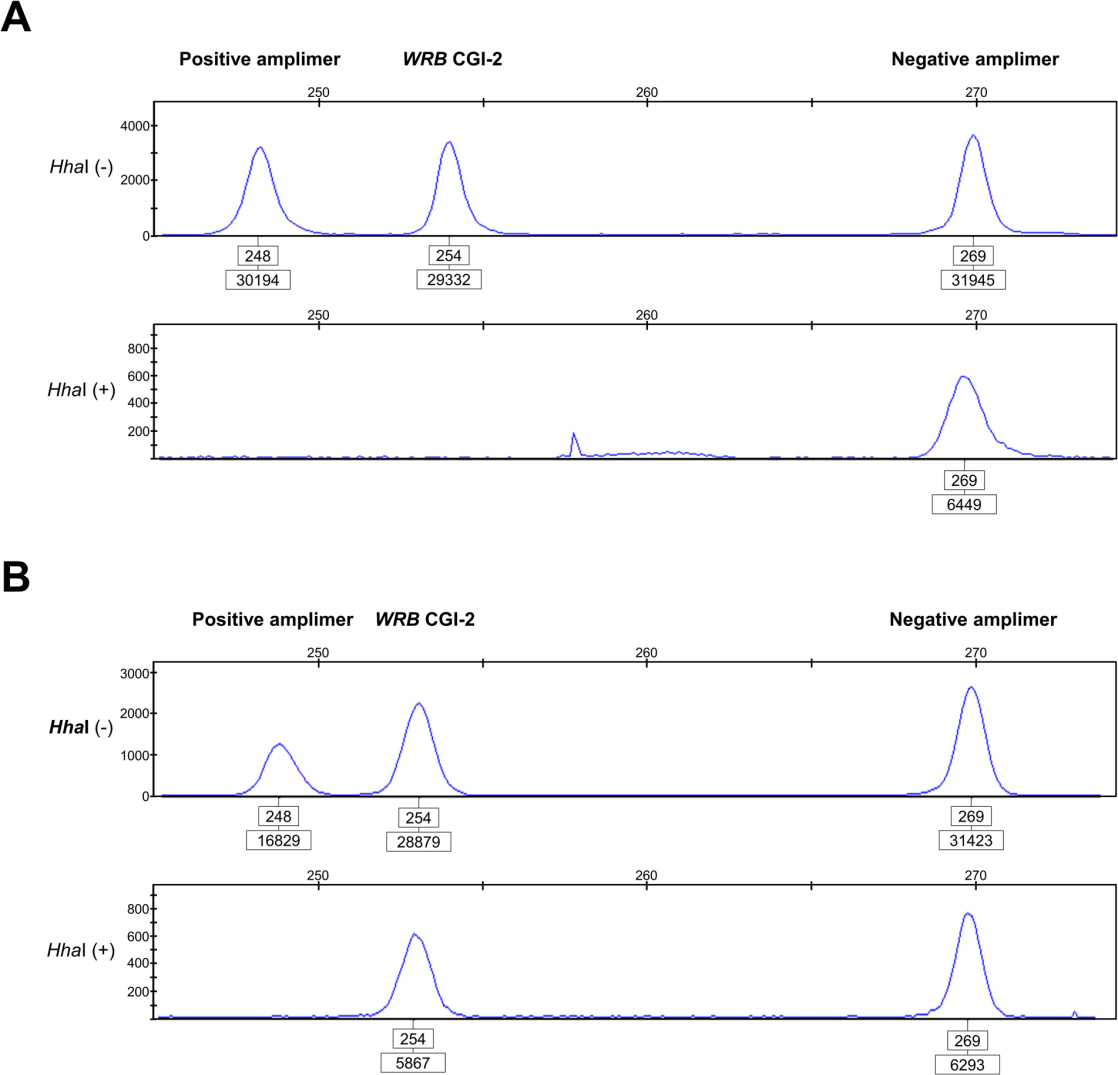
5^m^CpG statuses at the *WRB* CGI-2 DMR in a complete androgenetic mole and a human embryonic stem cell line. A consistent unmethylated pattern of CpG sites at the *WRB* CGI-2 DMR revealed in a sample of an androgenetic complete hydatidiform mole (**A**) contrast with the hypermethylated pattern observed in the representative HUES 3 embryonic cell line (**B**). Electropherograms of the amplimers (see details of the assay in Fig 2) generated from either undigested DNA or *Hha*I-digested DNA. The numbers in the upper boxes correspond to the amplimer lengths in base pairs while those in the lower boxes refer to the areas under the peak of the amplimer.

The ratios of restriction enzyme-resistant 5^m^CpG sites at the *WRB* CGI-2 DMR were significantly different between the MT21 probands (average ratio of 68.4%) and the PT21 probands (average ratio of 33.5%) (Fig 4 and S7 Table). As it follows from the inheritance of maternal-allele derived 5^m^CpG imprints, the *WRB* CGI-2 was unmethylated in DNA from a complete androgenetic mole (Figs 4 and 5A). In hESCs (Figs 4 and 5B, and S7 Table), the *WRB* CGI-2 was hypermethylated, indicating the loss of the imprinted pattern of 5^m^CpG marks at this developmental stage.

### Parent-of-Origin-Independent Altered Methylation Effects Due to the Extra Copy of Chromosome 21

**Fig 6:**
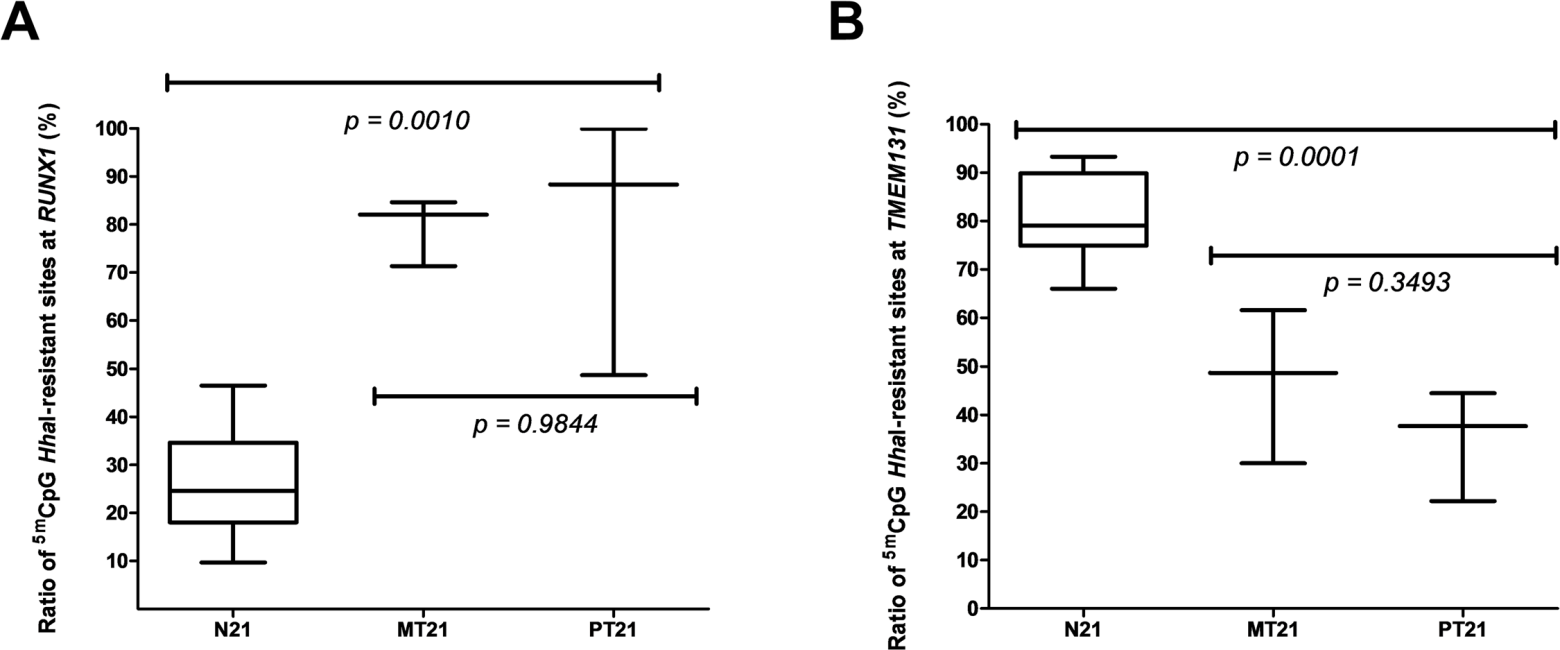
Parent-of-origin-independent methylation effects of the supernumerary chromosome 21 on both syntenic and non-syntenic genes. (**A**) A statistically significant gain of methylation at CpG dinucleotide sites within the syntenic *RUNX1* gene is observed in trisomic individuals compared with control disomic samples (N21). The gain is independent of the maternal (MT21) or the paternal (PT21) origin of the nondisjunction event. (**B**) A statistically significant loss of methylation at CpG dinucleotide sites within the non-syntenic *TMEM131* gene is observed in trisomic individuals compared with control disomic samples (N21). The loss is independent of the maternal (MT21) or the paternal (PT21) origin of the nondisjunction event.

Because the supernumerary copy of chromosome 21 differentially affects the methylation statuses at distinct *loci* in the genome [21, 22], we tested whether the levels of methylation at *loci* in either *cis-* or *trans-*configuration with regards to the *WRB* CGI-2 DMR change in a parent-of-origin-dependent fashion. Using the MSRE-PCR approach, we assessed the methylation statuses at CpG sites within the *RUNX1* (*cis*) and *TMEM131* (*trans*) genes that are differentially epigenetically perturbed in trisomic samples *versus* disomic samples [21, 22, 28]. Compared with disomic individuals, in trisomic subjects there is a gain of methylation at *RUNX1* and a loss of methylation at *TMEM131*. We replicated the reported perturbation effects of the extra copy of chromosome 21 on the methylation patterns at these *loci*. Importantly, and in contrast to the situation at the *WRB* CGI-2 DMR, the epigenetic perturbations observed at the *RUNX1* (Fig 6A) and *TMEM131* (Fig 6B) genes were independent of the parental origin of the supernumerary chromosome 21.

### The Maternal 5^m^CpG Imprints at the *WRB* CGI-2 DMR Do Not Dictate a Paternal Monoallelic Expression

**Fig 7:**
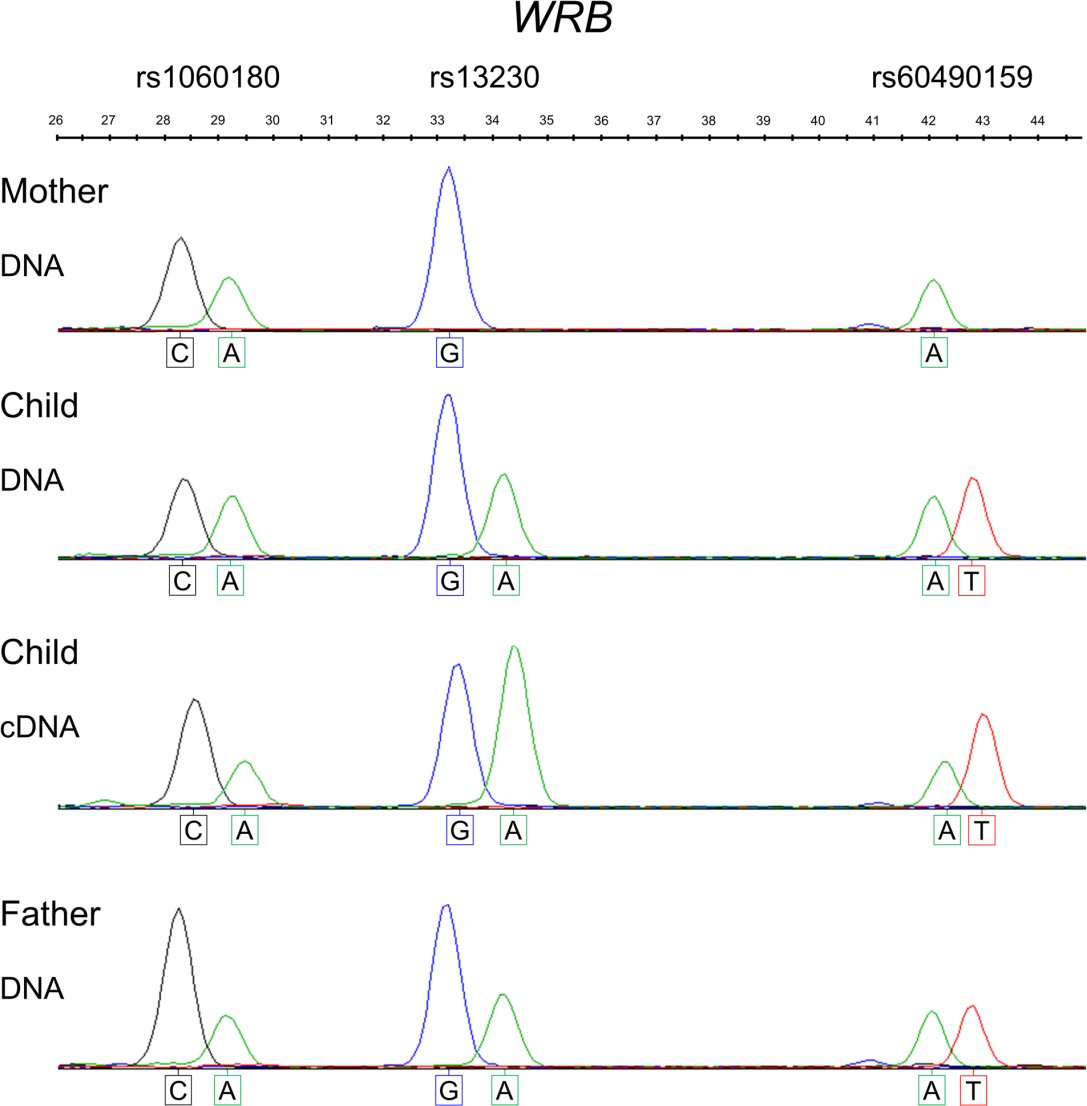
The maternally derived, imprinted 5^m^CpG marks at the *WRB* CGI-2 DMR do not dictate a paternal monoallelic expression in blood. Qualitative SNuPE allele-specific profiling of the *WRB* 3´-UTR rs1060180, rs13230 and rs60490159 SNPs in a disomic, informative nuclear family (DNA). The assay reveals a pattern consistent with biallelic transcriptional expression (cDNA) in the child, who is heterozygous for all three SNP variants.

**Fig 8:**
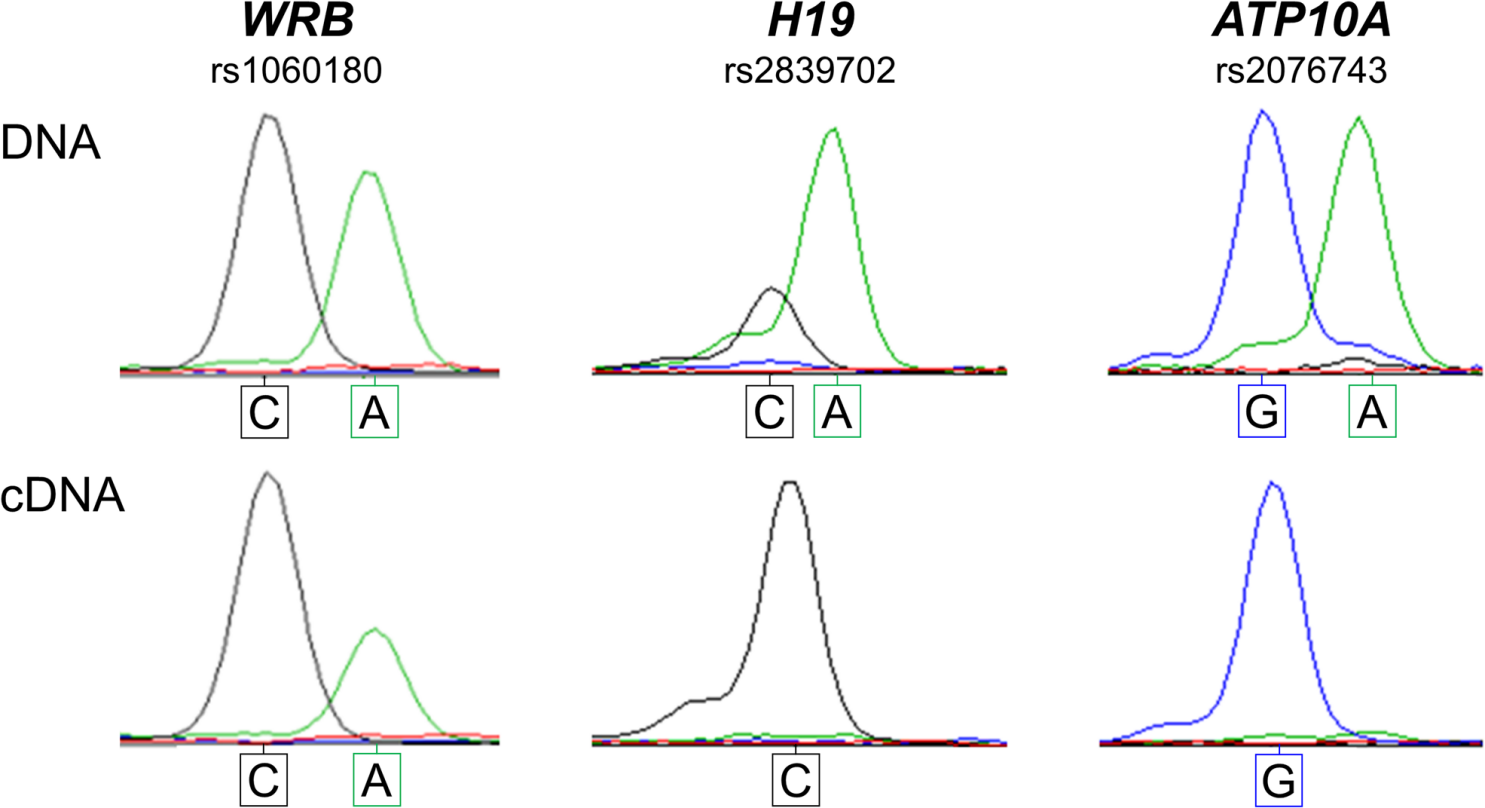
The maternally derived, imprinted 5^m^CpG marks at the *WRB* CGI-2 DMR do not dictate a paternal monoallelic expression in a human embryonic stem cell line. Allele-specific transcriptional profiling of the *WRB* 3´-UTR rs1060180 SNP in the informative hESC HUES 1 sample (DNA; upper panel) reveals a pattern consistent with biallelic expression (cDNA; lower panel). In contrast, for the known paternally imprinted *H19* and *ATP10A* genes, the expression profiles for the informative rs2839702 and rs2076743 SNPs, respectively, are monoallelic.

To assess whether the maternal allele-specific methylation pattern observed at the intragenic *WRB* CGI-2 DMR is indicative of functional gene regulation differences in the form of genomic imprinting, we qualitatively tested for allele-specific gene RNA expression by interrogating 3´-UTR SNP variants in cDNAs of RNA from blood samples and hESCs. We noted that both the maternal and paternal alleles were expressed in heterozygous samples independently of the methylation statuses observed at the *WRB* CGI-2 DMR (Fig 7). In hESCs, in which the *WRB* CGI-2 DMR is hypermethylated, the *WRB* transcriptional expression profile was biallelic (Fig 8).

Genomic imprinting can either completely silence one parental allele or significantly reduce its expression. We queried RNA-Seq public archives using sequence substrings for each exon 1 of the two major *WRB* reference transcripts, variants 1 (ENST00000333781.8) and 2 (ENST00000380708.4). We found evidence of expression (> 80 reads) of the *WRB* transcript variant 1 in RNA-Seq experiments in the brain, fallopian tube, liver, muscle, ovary, skin, and testis samples (S8 Table). However, using a sequence substring specific for the exon 1 of the *WRB* transcript variant 2, the number of reads filtered in each SRA accession from primary tissues was consistently ≤ 16, thus impairing further analysis of this short transcript variant (S8 Table) according to our stringent criteria.

Because relevant genotypes are unfortunately lacking in the entire RNA-Seq datasets analyzed (i.e., samples are not DNA/RNA exome sequencing pairs), we next aimed to provide RNA-Seq evidence for either monoallelic or biallelic expression queried with the rs1060180 and rs13230 *WRB* 3´-UTR SNPs. These SNPs map to both the long and short *WRB* transcript variants. In the 1,012 unsorted RNA-Seq experiments, we found allele fractions consistent with a biallelic pattern of expression (mean allele fraction ranging from 0.49 to 0.51) for both SNPs in the brain, epidermal keratinocytes, fetal large and small intestine, large airway epithelial cells, ovary, skin and testis samples (S9 Table). In the analysis of the RNA-Seq public databases sorted by tissue, we also observed allele fractions consistent with biallelic expression across the *WRB* 3´-UTR SNPs in brain, fallopian tube, thyroid, muscle, ovary, skin, and testis samples (S10 Table). Altogether, we observed no suppression or expression bias effects on either allele (i.e., the evidence against a maternally suppressed, imprinting effect) in ten biosamples (brain, fallopian tube, fetal large and small intestine, large airway epithelial cells, thyroid muscle, epidermal keratinocytes, ovary, skin, and testis). In contrast, for the known imprinted *SNURF* gene (tested across the rs705 SNP in the brain, fallopian tube, liver, muscle, ovary, skin, and testis) both alleles were expressed monoallelically (i.e., passed the “flip test” required for genomic imprinting) (S10 Table). Similarly, for the *H19* rs217727 and *H19* rs10840159 SNPs we observed monoallelic expression of both alleles in the fallopian tube, ovary, and testis (S10 Table).

**Fig 9:**
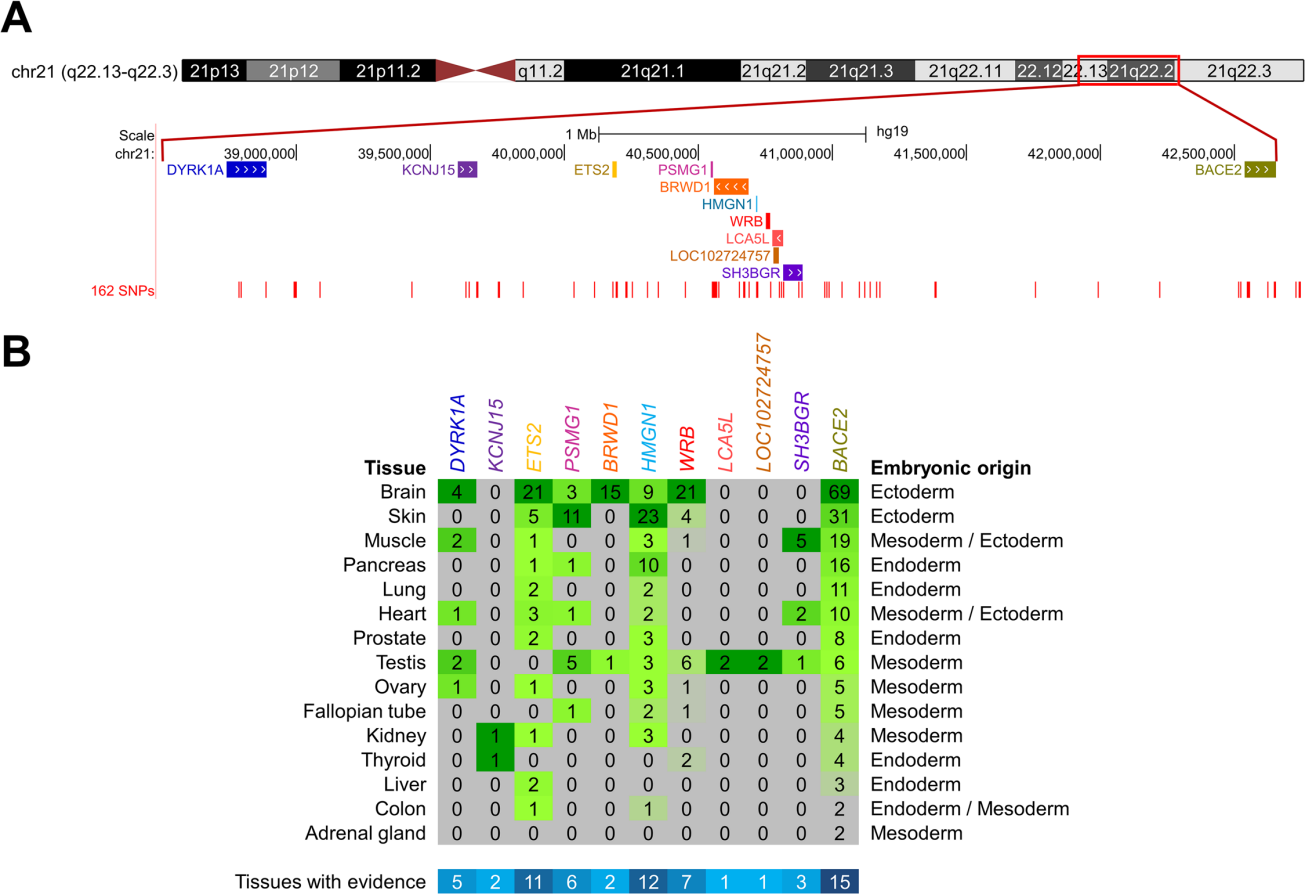
The allele expression of *WRB* and ten neighboring genes is uncoupled from the control of the maternally inherited 5^m^CpG imprints at the *WRB* DMR. (**A**) UCSC Genome Browser screenshot of custom tracks for the chromosomal and physical map positions of (top to bottom) the 11 genes that map within the 4-Mb chromosomal region centered at the *WRB* gene, for which evidence for biallelic transcriptional expression was unveiled in this study, and the relative locations of the 162 SNPs queried in RNA-seq public repositories for the determination of allele expression fractions. (**B**) Density distribution plot, by primary tissue, of the number of SRA accessions of RNA-seq experiments that yielded evidence consistent with biallelic expression. For each gene column, the intensity of the green color of each cell is proportional to the indicated numbers of informative SRA experiments. For each gene, the number of primary tissues with evidence is represented in the bottom row in blue scale.

Because some known DMRs are physically distant up to 2-Mb from the target imprinted gene [24, 25], and therefore function as imprinted control regions (ICRs), we extended the query to 162 SNPs (S4 Table), mapping within a 4-Mb chromosomal region centered at the *WRB* gene. Most SNP substrings yielded < 20 reads and, therefore, were unsuitable for further analysis according to the inclusion criteria. Importantly, in addition to *WRB*, we found SNPs with > 80 reads yielding allele fractions that were consistent with the biallelic expression in 15 biosamples in 10 genes: *DYRK1A*, *KCNJ15*, *ETS2*, *PSMG1*, *BRWD1*, *HMGN1*, *LOC102724757*, *LCA5L*, *SH3BGR*, and *BACE2* (Fig 9, and S10 and S11 Tables).

### Common Combinatorial Patterns of Histone Acetylation and Methylation Marks at the Predicted *WRB* Promoter Regions

**Fig 10:**
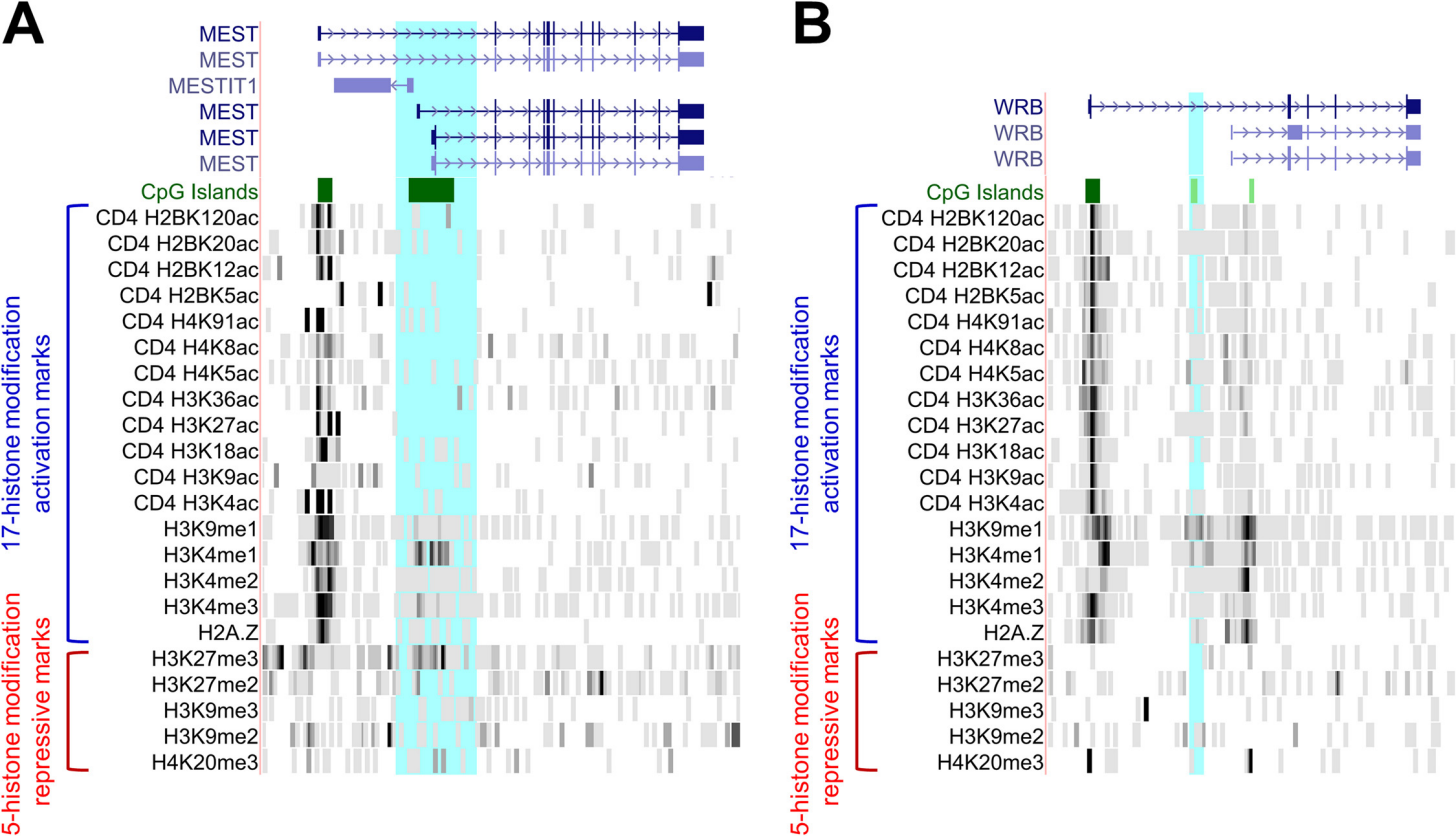
Common combinatorial histone modification expression patterns around the *WRB* CGI-2 DMRs. Graphical representation of the confluence of activating and repressive epigenetics histone modification marks for the known imprinted *MEST* gene (**A**) and the candidate imprinted *WRB* gene (**B**). Shown are the 17-histone modification activation backbone module and the 5-histone modification repressive module found in human CD4+ T cells [54]. Highlighted in light blue is the DMR in each gene. Composite of screenshots of the dataset viewed at the UCSC Genome Browser hg18 (http://genome.ucsc.edu).

We next intended to identify epigenetic histone marks around the *WRB* CGI-2 DMR that conflate traceable combinatorial patterns, which possibly regulate the expression patterns of the *WRB* transcript variants 1 and 2. We conducted the comparative analysis of DMRs classified as germline DMRs (gDMRs) [46] and associated with patterns of imprinted methylation or expression. We found similarities in the confluence of both the 17-histone modification activation backbone module and the 5-histone modification repressive module at the CGIs mapping to the long (ENST00000333781.8) and short (ENST00000380708.4) transcript variants of the *WRB* gene and at the gDMRs of the *BLCAP*, *GNAS*, *IGF2R*, *GRB10*, and *RB1* genes, which were most striking with the *MEST* gDMR (Fig 10 and S1 Fig).

### DNA Motifs at the *WRB* CGI-2 DMR

**Fig 11:**
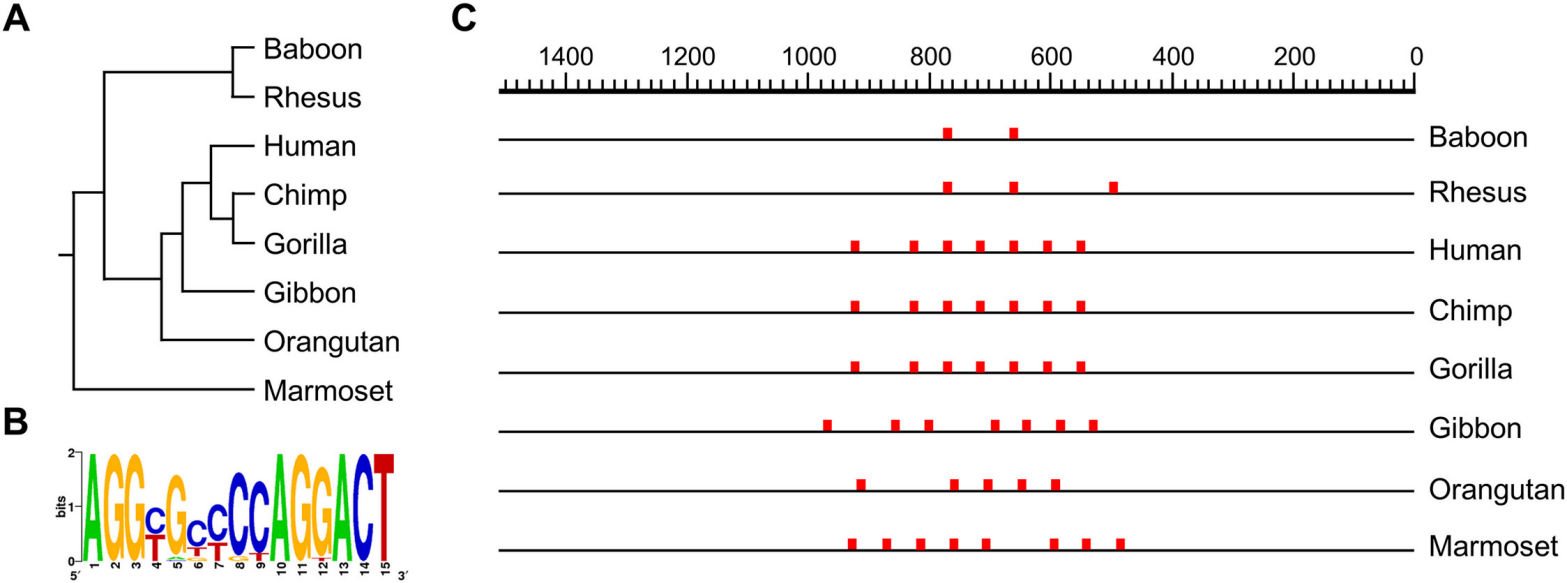
The *WRB* CGI-2 DMR contains a primate-conserved cluster array of a DNA motif. (**A**) Molecular phylogenetic relationship of eight *WRB* CGI-2 DNA primate reference sequences inferred using multiple sequence alignment by CLUSTALW. (**B**) Sequence logo of the primate-conserved [AGGYGBYSYAGGACT] DNA repeat unit that occurs at the *WRB* CGI-2. (**C**) Physical map of the primate-conserved [AGGYGBYSYAGGACT] DNA repeat unit array.

Based on a computational comparative analysis of genomic reference sequences, we observed that the nucleotide sequence encompassing the CGI-2 is conserved among primates (Fig 11A). We analyzed the region for the occurrence of arrays of *cis* elements (other than the CpG dinucleotide sites) that may be specific to this DMR or common to known DMRs by searching for sequence motifs as testable predictions of the differential epigenetic status. We found the DNA repeat motif [AGGYGBYSYAGGACT] (Fig 11B). In humans, the motif occurs in a cluster of seven tandem units. The motif repeat units are spaced at regular chromosomal intervals through the *WRB* DMR (CGI-2), encompassing 388 bp (Fig 11C). We then searched the whole genome reference sequence and were unable to find other *loci* containing the array that occurs at the *WRB* CGI-2 DMR. The [AGGYGBYSYAGGACT] motif repeat unit has sequence similarity (i.e., match overlap of thirteen nucleotides in the optimal alignment) to the putative multimer SPDEF_DBD_2 DNA-binding specificity consensus site ([gtggTCCCGGATYAT]) of the transcriptional factor SPDEF (HumanTF 1.0 DNA binding motif library) [49, 63]. The number of [AGGYGBYSYAGGACT] DNA motif units has varied since its evolutionary occurrence in marmosets (Fig 11C).

## Discussion

In this multidisciplinary study, we used complementary experimental and computational approaches to address the challenging biological questions of whether the extra copy of chromosome 21 in Down syndrome affects the methylation patterns at distinct CpG dinucleotide sites and whether the epigenetic alterations occur dependently or independently of the parental origin of the nondisjoined chromosome 21.

Although prior studies had shown that the extra copy of chromosome 21 differentially affects the DNA methylation levels at distinct CpG dinucleotide sites [21, 22], it was unknown whether the effects are dependent (i.e., genomic imprinting) and/or independent of the parental origin of the nondisjoined chromosome 21. We showed these epigenetic effects on three genes, two located on chromosome 21 (*WRB* and *RUNX1*) and one located on chromosome 2 (*TMEM131*).

We established that the supernumerary chromosome 21 altered the methylation patterns at distinct CpG dinucleotide sites in the *RUNX1* and *TMEM131* genes in a parent-of-origin-independent manner. From a genome-wide perspective, this finding suggests that the epigenetic effect of the extra copy of chromosome 21 does not vary greatly with the parental origin of the supernumerary copy of chromosome 21; however, the extra copy does affect the methylation statuses of genes located on the same and other chromosomes.

We explored the differentially heritable epigenetic methylation imprints at the *WRB* CGI-2 DMR to develop a simple PCR assay based on the maternally imprinted 5^m^CpG marks to ascertain the parental origin of chromosome 21 nondisjunctional events in Down syndrome probands. The assay does not require bisulfite conversion and improves on the current linkage analysis approach by not requiring genomic DNA from the progenitors. This epigenetic feature will be an asset when parental samples are unavailable as in the situation of cryopreserved banked specimens. In addition to the notorious scarcity (incidence of approximately 5%) of the nondisjunctional events of paternal origin, the dependability of their identification from DNA available from the progenitors has precluded studies on phenotype-(epi)genotype correlations for Down syndrome of patrilineal descent. The *WRB* CGI-2 DMR-based assay will greatly facilitate the identification of Down syndrome cases of paternal origin and the establishment of representative cohorts for studies on the variation in phenotypic outcomes. The assay will also be useful in the prenatal molecular diagnosis of the parental origin, without collecting parental samples, in triploid pregnancies where only the conceptuses with two paternal sets have the potential to cause maternal complications [64].

The initial evidence suggesting a functional link between the maternal-of-origin-specific imprinted methylation at the *WRB* CGI-2 DMR and the *WRB* monoallelic expression came from the study by Docherty and collaborators [25]. By sequencing cDNA across the rs1060180 SNP, they observed alternate (i.e., maternal or paternal) monoallelic transcript expression in skeletal muscle and aorta tissues and biallelic transcript expression in the spinal cord from the same embryo. Unfortunately, that study did not conclusively establish whether the paternal allele is unmethylated. Moreover, a distinctive feature of imprinted genes is the preference for the full expression of one (and always the same) of the two parental alleles [65]. Importantly, alternate monoallelic transcript expression occurs only between different genes regulated by the same imprinting control region. For example, between the paternally expressed *Peg13* non-coding RNA and the maternally expressed *Kcnk9* gene in the mouse brain [66], and between the maternally expressed *H19* and the paternally expressed *Igf2* genes in mouse hematopoietic stem and progenitor cells [67].

We evaluated the candidate imprinting status of the *WRB* gene. We showed that the maternal heritable epigenetic 5^m^CpG imprints at a CGI-2 DMR were uncoupled to the predicted monoallelic expression of the paternal *WRB* allele. We replicated this observation in twelve biosamples (brain, blood, fallopian tube, fetal large and small intestine, hESCs, large airway epithelial cells, thyroid, muscle, epidermal keratinocytes, ovary, skin, and testis). These results are in agreement with recently reported findings from three genome-wide scale analyses of public RNA-Seq data [26, 59, 68]. These studies reported mean allele fractions showing either non-significant deviation (mean value = 0.504) [26, 59] or significant deviation (average value ranging from 0.553 to 0.803) [26, 68] for the *WRB* 3´-UTR rs1060180 and rs13230 SNPs. Furthermore, we provide evidence from 15 tissue repositories of allele rates that are consistent with the biallelic transcript expression of 10 genes that map to within 2-Mb around the *WRB* gene. Thus, the allele transcript expression of 11 genes in at least one primary human tissue tested is uncoupled from the control of maternally inherited imprints at the *WRB* DMR.

Steyaert and collaborators [26] used a SNP-guided analytical framework to identify monoallelic DNA methylation events from enrichment-based sequencing data. In the *WRB* gene, they functionally annotated the *locus* comprising the SNP rs2299739 with significant monoallelic DNA methylation. Using the rs2244352 SNP, here we demonstrated that the *locus* undergoes maternal-of-origin-specific differential methylation. We showed that in hESCs the *WRB* CGI-2 is hypermethylated, in contrast to the differential maternally inherited allele methylation profile observed in blood cells. A hypermethylated state for the *WRB* CGI-2 DMR occurs in several methylome studies of hESCs [35, 41, 42]. Significantly, we observed a biallelic transcriptional expression pattern for *WRB*, despite the hypermethylated state in hESCs.

The hemimethylated status characteristic of the known imprinted DMRs is not a sufficient epigenetic signature to determine the uniparental monoallelic expression of genes. For example, the gDMR in the *GRB10* gene, which is isoform-specifically imprinted only in the brain, is hemimethylated in all tissues analyzed [55, 69]. We observed a comparable confluence of both the 17-histone modification activation backbone module and the 5-histone modification repressive module between six maternally imprinted genes (*MEST*, *BLCAP*, *GNAS*, *IGF2R*, *GRB10*, and *RB1*) and the *WRB* gene. Therefore, there is still the possibility of the *WRB* gene being an isoform- and tissue-specific imprinted gene. Due to the unavailability of isoform-specific SNPs and the small levels of expression of the *WRB* transcript variant 2 in RNA-Seq experiments, unfortunately, we could not test that hypothesis with our current approach.

In a recent methylome study in human oocytes, Okae and collaborators [46] used the criteria of DNA methylation levels at the DMRs of known imprinted genes to classify the *WRB* CGI-2 as a secondary DMR (i.e., rather than a gDMR). At the *WRB* CGI-2, they observed an average methylation of 17.5% in oocytes (hg19 coordinates chr21:40757510–40758276; 11.5% in oocytes pool 1 PCR and 49.9% in oocyte pool 2) and of 0.45% in sperm, but with 35–65% methylation levels in blood cells. Importantly, a genome-wide scan of the methylomes of oocytes and blastocysts from that study revealed that the methylation profiles are highly comparable, with the methylation levels of maternal germline DMRs in blastocysts being on average half the levels found in oocytes (data not shown). Because the reported level of methylation at the *WRB* CGI-2 in blastocyst was 37.72% [46], one would expect a methylation level of approximately 75% in oocytes. In fact, the average methylation level at a core region with hg19 coordinates chr21: 40757603–40757721 in oocyte pool 2 is 70.3% (reanalysis of data from S1 Table of ref. [46]). Therefore, we consider the *WRB* CGI-2 to be a maternal gDMR.

We demonstrated the maintenance of the DNA sequence context of the human *WRB* CGI-2 DMR in primates. Within the *WRB* CGI-2 DMR, we identified a *cis* DNA motif array. In primates, the array consists of 2 to 8 consensus repeat units in primates that bear sequence similarity to the DNA-binding specificity consensus site of the transcription factor SPDEF. We speculate that the *cis* DNA motif array is a distinctive feature in the establishment and/or maintenance of the hemimethylated state of this particular DMR, rather than being involved in the differential expression of *WRB* transcript variants or alleles.

*WRB* is essentially a housekeeping gene. The human WRB protein corresponds to the Get1 protein in yeast. The WRB/Get1 proteins form a conserved family (IPR028945) in eukaryotes. These proteins function as transmembrane receptors for ASNA1/TRC40 (Get3 in yeast)-mediated insertion of tail-anchored (TA) proteins into the endoplasmic reticulum membrane (GO:0071816) [70]. RNA-Seq profiling revealed that *WRB* transcription occurs in all individual tissue categories investigated, although higher levels (i.e., FPKM/TPM) are displayed in the brain, testis, ovary and kidney [71]. Most tissues displayed moderate to strong nuclear and cytoplasmic positivity by WRB-specific antibody staining [72]. RNAi-mediated knockdown of *WRB* expression increases the rate of homologous recombination DNA double-strand break repair [73]. Importantly, *WRB* transcription levels are compensated in trisomic T21 lymphoblastoid cells [74], and the *WRB* gene lies outside a chromosomal domain dysregulated by the presence of the supernumerary chromosome 21 [20]. Therefore, the biological significance of the *WRB* gene perhaps being imprinted in an isoform, tissue and/or developmental-stage-specific manner is rather intriguing.

## Supporting Information

S1 Fig Comparison of the common combinatorial histone modification expression patterns around the *WRB* CGI-2 DMRs and known imprinted genes. Graphical representation of the confluence of activating and repressive epigenetics histone modification marks for the known imprinted genes (**A**) *BLCAP*, (**B**) *GNAS*, (**C**) *IGF2R*, (**D**) *GRB10* and (**E**) *RB1*. Shown are the 17-histone modification activation backbone module, and the 5-histone modification repressive module found in human CD4+ T cells [54]. Highlighted in light blue is the DMR in each gene. Composite of screenshots of the dataset viewed at the UCSC Genome Browser hg18 (http://genome.ucsc.edu).
(TIF)

S1 Table List of target *loci*, primer sequences, and restriction enzymes interrogated for parent-of-origin- dependent and -independent effects on DNA methylation. (XLSX)

S2 Table STR genotypes and allele segregation analysis used in the parental origin determination of the extra chromosome 21 in trisomy 21 nuclear families. (XLSX)

S3 Table STR genotypes and allele segregation analysis used in the genetic characterization of the androgenetic complete hydatidiform mole (ACHM) and the peripheral blood of the donor. (XLSX)

S4 Table Consolidated information regarding the 162 SNPs that map within the 4-Mb chromosomal region centered at the *WRB* gene queried in public RNA-Seq repositories of fifteen primary tissues to determine the status of allele expression. (XLS)

S5 Table List of the 1,012 RNA-Seq public datasets queried to investigate *locus* and allele-specific expression. The list is a manually curated compilation of tables downloaded from the NCBI Sequence Read Archive (SRA).
(XLSX)

S6 Table List of the RNA-Seq public data experiments configuring the Atlas of primary human tissues queried to replicate the imprinting-dependent and independent patterns of allele-specific expression of target genes. The list is a manually curated compilation of tables downloaded from the NCBI Sequence Read Archive (SRA), Biosample and Bioproject browsers.
(XLS)

S7 Table Ratios of the restriction enzyme-resistant 5^m^CpG sites in the *WRB* CGI-2 estimated in control disomic and trisomic study subjects and in human embryonic stem cell lines. (XLSX)

S8 Table RNA-Seq reads filtered with sequence substrings specific for each exon 1 of either *WRB* transcript variants 1 (ENST00000333781.8, long) or 2 (ENST00000380708.4, short). (XLS)

S9 Table Allele expression fractions across the *WRB* 3´-UTR rs1060180 and rs13230 SNPs found using unsorted 1,012 RNA-Seq public datasets. (XLSX)

S10 Table Allele expression fractions across the 162 SNPs mapping within the 4-Mb chromosomal region centered at the candidate imprinted *WRB* gene and across SNPs in the *SNURF* and *H19* known imprinted genes. Shown in the different worksheets are the number of reads for each SNP filtered in the RNA-Seq public datasets, sorted by informative tissue. Worksheet labels correspond to the series “tissue gene SNP” (i.e., Adrenal *BACE2* rs11701157), grouped alphabetically, and highlighted in different colors by tissue.
(XLS)

S11 Table Summary of the RNA-seq evidence of biallelic expression of eleven genes mapping to a 4-Mb chromosomal region centered at the *WRB* gene in fifteen primary human tissues (Fig 9 in the main text is a graphical representation of these data). (XLS)

## References

[1] Megarbane A, Ravel A, Mircher C, Sturtz F, Grattau Y, Rethore MO, et al The 50th anniversary of the discovery of trisomy 21: the past, present, and future of research and treatment of Down syndrome. Genet Med. 2009;11(9):611–6. 10.1097/GIM.0b013e3181b2e34c . 19636252

[2] Antonarakis SE. Parental origin of the extra chromosome in trisomy 21 as indicated by analysis of DNA polymorphisms. Down Syndrome Collaborative Group. N Engl J Med. 1991;324(13):872–6. 10.1056/NEJM199103283241302 . 1825697

[3] Oliver TR, Feingold E, Yu K, Cheung V, Tinker S, Yadav-Shah M, et al New insights into human nondisjunction of chromosome 21 in oocytes. PLoS Genet. 2008;4(3):e1000033 10.1371/journal.pgen.1000033 18369452 PMC2265487

[4] Gaulden ME. Maternal age effect: the enigma of Down syndrome and other trisomic conditions. Mutat Res. 1992;296(1–2):69–88. . 1279409 10.1016/0165-1110(92)90033-6

[5] Erickson JD, Bjerkedal TO. Down syndrome associated with father's age in Norway. J Med Genet. 1981;18(1):22–8. 6454784 10.1136/jmg.18.1.22 PMC1048652

[6] Hatch M, Kline J, Levin B, Hutzler M, Warburton D. Paternal age and trisomy among spontaneous abortions. Hum Genet. 1990;85(3):355–61. . 2394449 10.1007/BF00206761

[7] Kazaura MR, Lie RT. Down's syndrome and paternal age in Norway. Paediatr Perinat Epidemiol. 2002;16(4):314–9. . 12445147 10.1046/j.1365-3016.2002.00446.x

[8] De Souza E, Morris JK, Group EW. Case-control analysis of paternal age and trisomic anomalies. Arch Dis Child. 2010;95(11):893–7. 10.1136/adc.2009.176438 . 20584846

[9] Zigman WB. Atypical aging in Down syndrome. Dev Disabil Res Rev. 2013;18(1):51–67. 10.1002/ddrr.1128 . 23949829

[10] Stoll C, Alembik Y, Dott B, Feingold J. No evidence for genomic imprinting in liver-born Down syndrome patients. Acta Genet Med Gemellol (Roma). 1996;45(1–2):265–71. 8872044 10.1017/s0001566000001434

[11] Muranjan M, Chaudhari T, Vundinti BR. Phenotypic heterogeneity and parental origin of extra chromosome 21 in Down syndrome. Indian Pediatr. 2010;47(5):429–32. . 19671944 10.1007/s13312-010-0078-2

[12] Henderson DJ, Sherman LS, Loughna SC, Bennett PR, Moore GE. Early embryonic failure associated with uniparental disomy for human chromosome 21. Hum Mol Genet. 1994;3(8):1373–6. . 7987317 10.1093/hmg/3.8.1373

[13] Creau-Goldberg N, Gegonne A, Delabar J, Cochet C, Cabanis MO, Stehelin D, et al Maternal origin of a de novo balanced t(21q21q) identified by ets-2 polymorphism. Hum Genet. 1987;76(4):396–8. . 2886422 10.1007/BF00272452

[14] Rogan PK, Sabol DW, Punnett HH. Maternal uniparental disomy of chromosome 21 in a normal child. Am J Med Genet. 1999;83(1):69–71. . 10076888 10.1002/(sici)1096-8628(19990305)83:1<69::aid-ajmg14>3.0.co;2-q

[15] Mansuet-Lupo A, Henke J, Henke L, Blank C, Ernsting A, Kozlowski P, et al A paternity case with three genetic incompatibilities between father and child due to maternal uniparental disomy 21 and a mutation at the Y chromosome. Forensic Sci Int Genet. 2009;3(2):141–3. 10.1016/j.fsigen.2008.09.010 . 19215885

[16] Blouin JL, Avramopoulos D, Pangalos C, Antonarakis SE. Normal phenotype with paternal uniparental isodisomy for chromosome 21. Am J Hum Genet. 1993;53(5):1074–8. 8213833 PMC1682298

[17] Robinson WP, Bernasconi F, Basaran S, Yuksel-Apak M, Neri G, Serville F, et al A somatic origin of homologous Robertsonian translocations and isochromosomes. Am J Hum Genet. 1994;54(2):290–302. 8304346 PMC1918173

[18] Liehr T. Uniparental Disomy (UPD) in Clinical Genetics. Berlin: Springer Heidelberg 2014. 201 p.

[19] Conti A, Fabbrini F, D'Agostino P, Negri R, Greco D, Genesio R, et al Altered expression of mitochondrial and extracellular matrix genes in the heart of human fetuses with chromosome 21 trisomy. BMC Genomics. 2007;8:268 10.1186/1471-2164-8-268 17683628 PMC1964766

[20] Letourneau A, Santoni FA, Bonilla X, Sailani MR, Gonzalez D, Kind J, et al Domains of genome-wide gene expression dysregulation in Down's syndrome. Nature. 2014;508(7496):345–50. 10.1038/nature13200 . 24740065

[21] Kerkel K, Schupf N, Hatta K, Pang D, Salas M, Kratz A, et al Altered DNA methylation in leukocytes with trisomy 21. PLoS Genet. 2010;6(11):e1001212 10.1371/journal.pgen.1001212 21124956 PMC2987931

[22] Jones MJ, Farre P, McEwen LM, Macisaac JL, Watt K, Neumann SM, et al Distinct DNA methylation patterns of cognitive impairment and trisomy 21 in Down syndrome. BMC Med Genomics. 2013;6:58 10.1186/1755-8794-6-58 24373378 PMC3879645

[23] Oliver TR, Bhise A, Feingold E, Tinker S, Masse N, Sherman SL. Investigation of factors associated with paternal nondisjunction of chromosome 21. Am J Med Genet A. 2009;149A(8):1685–90. 10.1002/ajmg.a.32942 19606484 PMC4111419

[24] Court F, Tayama C, Romanelli V, Martin-Trujillo A, Iglesias-Platas I, Okamura K, et al Genome-wide parent-of-origin DNA methylation analysis reveals the intricacies of human imprinting and suggests a germline methylation-independent mechanism of establishment. Genome Res. 2014;24(4):554–69. 10.1101/gr.164913.113 24402520 PMC3975056

[25] Docherty LE, Rezwan FI, Poole RL, Jagoe H, Lake H, Lockett GA, et al Genome-wide DNA methylation analysis of patients with imprinting disorders identifies differentially methylated regions associated with novel candidate imprinted genes. J Med Genet. 2014;51(4):229–38. 10.1136/jmedgenet-2013-102116 24501229 PMC3963529

[26] Steyaert S, Van Criekinge W, De Paepe A, Denil S, Mensaert K, Vandepitte K, et al SNP-guided identification of monoallelic DNA-methylation events from enrichment-based sequencing data. Nucleic Acids Res. 2014;42(20):e157 10.1093/nar/gku847 25237057 PMC4227762

[27] Wang X, Clark AG. Using next-generation RNA sequencing to identify imprinted genes. Heredity (Edinb). 2014;113(2):156–66. 10.1038/hdy.2014.18 24619182 PMC4105452

[28] Bacalini MG, Gentilini D, Boattini A, Giampieri E, Pirazzini C, Giuliani C, et al Identification of a DNA methylation signature in blood cells from persons with Down Syndrome. Aging (Albany NY). 2015;7(2):82–96. 25701644 10.18632/aging.100715 PMC4359691

[29] Sambrook J, Russell DW. Molecular cloning: a laboratory manual 3rd ed. Cold Spring Harbor: Cold Spring Harbor Laboratory Press; 2001. 999 p p.

[30] Machado FB, Machado FB, Faria MA, Lovatel VL, Alves da Silva AF, Radic CP, et al 5meCpG epigenetic marks neighboring a primate-conserved core promoter short tandem repeat indicate X-chromosome inactivation. PLoS One. 2014;9(7):e103714 10.1371/journal.pone.0103714 25078280 PMC4117532

[31] Sutherland E, Coe L, Raleigh EA. McrBC: a multisubunit GTP-dependent restriction endonuclease. J Mol Biol. 1992;225(2):327–48. . 1317461 10.1016/0022-2836(92)90925-a

[32] Rachmilewitz J, Goshen R, Ariel I, Schneider T, de Groot N, Hochberg A. Parental imprinting of the human H19 gene. FEBS Lett. 1992;309(1):25–8. . 1380925 10.1016/0014-5793(92)80731-u

[33] Meguro M, Kashiwagi A, Mitsuya K, Nakao M, Kondo I, Saitoh S, et al A novel maternally expressed gene, ATP10C, encodes a putative aminophospholipid translocase associated with Angelman syndrome. Nat Genet. 2001;28(1):19–20. 10.1038/88209 . 11326269

[34] Song Q, Decato B, Hong EE, Zhou M, Fang F, Qu J, et al A reference methylome database and analysis pipeline to facilitate integrative and comparative epigenomics. PLoS One. 2013;8(12):e81148 10.1371/journal.pone.0081148 24324667 PMC3855694

[35] Roadmap Epigenomics C, Kundaje A, Meuleman W, Ernst J, Bilenky M, Yen A, et al Integrative analysis of 111 reference human epigenomes. Nature. 2015;518(7539):317–30. 10.1038/nature14248 25693563 PMC4530010

[36] Kent WJ, Sugnet CW, Furey TS, Roskin KM, Pringle TH, Zahler AM, et al The human genome browser at UCSC. Genome Res. 2002;12(6):996–1006. 10.1101/gr.229102 Article published online before print in May 2002. 12045153 PMC186604

[37] Raney BJ, Dreszer TR, Barber GP, Clawson H, Fujita PA, Wang T, et al Track data hubs enable visualization of user-defined genome-wide annotations on the UCSC Genome Browser. Bioinformatics. 2014;30(7):1003–5. 10.1093/bioinformatics/btt637 24227676 PMC3967101

[38] Zeng J, Konopka G, Hunt BG, Preuss TM, Geschwind D, Yi SV. Divergent whole-genome methylation maps of human and chimpanzee brains reveal epigenetic basis of human regulatory evolution. Am J Hum Genet. 2012;91(3):455–65. 10.1016/j.ajhg.2012.07.024 22922032 PMC3511995

[39] Lister R, Mukamel EA, Nery JR, Urich M, Puddifoot CA, Johnson ND, et al Global epigenomic reconfiguration during mammalian brain development. Science. 2013;341(6146):1237905 10.1126/science.1237905 23828890 PMC3785061

[40] Ziller MJ, Gu H, Muller F, Donaghey J, Tsai LT, Kohlbacher O, et al Charting a dynamic DNA methylation landscape of the human genome. Nature. 2013;500(7463):477–81. 10.1038/nature12433 23925113 PMC3821869

[41] Xie W, Schultz MD, Lister R, Hou Z, Rajagopal N, Ray P, et al Epigenomic analysis of multilineage differentiation of human embryonic stem cells. Cell. 2013;153(5):1134–48. 10.1016/j.cell.2013.04.022 23664764 PMC3786220

[42] Lister R, Pelizzola M, Kida YS, Hawkins RD, Nery JR, Hon G, et al Hotspots of aberrant epigenomic reprogramming in human induced pluripotent stem cells. Nature. 2011;471(7336):68–73. 10.1038/nature09798 21289626 PMC3100360

[43] Li Y, Zhu J, Tian G, Li N, Li Q, Ye M, et al The DNA methylome of human peripheral blood mononuclear cells. PLoS Biol. 2010;8(11):e1000533 10.1371/journal.pbio.1000533 21085693 PMC2976721

[44] Hodges E, Molaro A, Dos Santos CO, Thekkat P, Song Q, Uren PJ, et al Directional DNA methylation changes and complex intermediate states accompany lineage specificity in the adult hematopoietic compartment. Mol Cell. 2011;44(1):17–28. 10.1016/j.molcel.2011.08.026 21924933 PMC3412369

[45] Heyn H, Li N, Ferreira HJ, Moran S, Pisano DG, Gomez A, et al Distinct DNA methylomes of newborns and centenarians. Proc Natl Acad Sci U S A. 2012;109(26):10522–7. 10.1073/pnas.1120658109 22689993 PMC3387108

[46] Okae H, Chiba H, Hiura H, Hamada H, Sato A, Utsunomiya T, et al Genome-wide analysis of DNA methylation dynamics during early human development. PLoS Genet. 2014;10(12):e1004868 10.1371/journal.pgen.1004868 25501653 PMC4263407

[47] Flicek P, Amode MR, Barrell D, Beal K, Billis K, Brent S, et al Ensembl 2014. Nucleic Acids Res. 2014;42(Database issue):D749–55. 10.1093/nar/gkt1196 24316576 PMC3964975

[48] Bailey TL, Boden M, Buske FA, Frith M, Grant CE, Clementi L, et al MEME SUITE: tools for motif discovery and searching. Nucleic Acids Res. 2009;37(Web Server issue):W202–8. 10.1093/nar/gkp335 19458158 PMC2703892

[49] Sebastian A, Contreras-Moreira B. footprintDB: a database of transcription factors with annotated cis elements and binding interfaces. Bioinformatics. 2014;30(2):258–65. 10.1093/bioinformatics/btt663 . 24234003

[50] Benson G. Tandem repeats finder: a program to analyze DNA sequences. Nucleic Acids Res. 1999;27(2):573–80. 9862982 10.1093/nar/27.2.573 PMC148217

[51] Whitaker JW, Chen Z, Wang W. Predicting the human epigenome from DNA motifs. Nat Methods. 2015;12(3):265–72, 7 p following 72. 10.1038/nmeth.3065 25240437 PMC4344378

[52] Gupta S, Stamatoyannopoulos JA, Bailey TL, Noble WS. Quantifying similarity between motifs. Genome Biol. 2007;8(2):R24 10.1186/gb-2007-8-2-r24 17324271 PMC1852410

[53] Grant CE, Bailey TL, Noble WS. FIMO: scanning for occurrences of a given motif. Bioinformatics. 2011;27(7):1017–8. 10.1093/bioinformatics/btr064 21330290 PMC3065696

[54] Wang Z, Zang C, Rosenfeld JA, Schones DE, Barski A, Cuddapah S, et al Combinatorial patterns of histone acetylations and methylations in the human genome. Nat Genet. 2008;40(7):897–903. 10.1038/ng.154 18552846 PMC2769248

[55] Baran Y, Subramaniam M, Biton A, Tukiainen T, Tsang EK, Rivas MA, et al The landscape of genomic imprinting across diverse adult human tissues. Genome Res. 2015;25(7):927–36. 10.1101/gr.192278.115 25953952 PMC4484390

[56] Barrett T, Edgar R. Gene expression omnibus: microarray data storage, submission, retrieval, and analysis. Methods Enzymol. 2006;411:352–69. 10.1016/S0076-6879(06)11019-8 16939800 PMC1619900

[57] Parkinson H, Sarkans U, Kolesnikov N, Abeygunawardena N, Burdett T, Dylag M, et al ArrayExpress update—an archive of microarray and high-throughput sequencing-based functional genomics experiments. Nucleic Acids Res. 2011;39(Database issue):D1002–4. 10.1093/nar/gkq1040 21071405 PMC3013660

[58] Smith RM, Webb A, Papp AC, Newman LC, Handelman SK, Suhy A, et al Whole transcriptome RNA-Seq allelic expression in human brain. BMC Genomics. 2013;14:571 10.1186/1471-2164-14-571 23968248 PMC3765493

[59] Deelen P, Zhernakova DV, de Haan M, van der Sijde M, Bonder MJ, Karjalainen J, et al Calling genotypes from public RNA-sequencing data enables identification of genetic variants that affect gene-expression levels. Genome Med. 2015;7(1):30 10.1186/s13073-015-0152-4 25954321 PMC4423486

[60] Bentley DR, Balasubramanian S, Swerdlow HP, Smith GP, Milton J, Brown CG, et al Accurate whole human genome sequencing using reversible terminator chemistry. Nature. 2008;456(7218):53–9. 10.1038/nature07517 18987734 PMC2581791

[61] Blankenberg D, Gordon A, Von Kuster G, Coraor N, Taylor J, Nekrutenko A, et al Manipulation of FASTQ data with Galaxy. Bioinformatics. 2010;26(14):1783–5. 10.1093/bioinformatics/btq281 20562416 PMC2894519

[62] Krueger F, Kreck B, Franke A, Andrews SR. DNA methylome analysis using short bisulfite sequencing data. Nat Methods. 2012;9(2):145–51. 10.1038/nmeth.1828 . 22290186

[63] Jolma A, Yan J, Whitington T, Toivonen J, Nitta KR, Rastas P, et al DNA-binding specificities of human transcription factors. Cell. 2013;152(1–2):327–39. 10.1016/j.cell.2012.12.009 . 23332764

[64] Joergensen MW, Rasmussen AA, Niemann I, Hindkjaer J, Agerholm I, Bolund L, et al Methylation-specific multiplex ligation-dependent probe amplification: utility for prenatal diagnosis of parental origin in human triploidy. Prenat Diagn. 2013;33(12):1131–6. 10.1002/pd.4206 . 23881788

[65] Proudhon C, Bourc'his D. Identification and resolution of artifacts in the interpretation of imprinted gene expression. Brief Funct Genomics. 2010;9(5–6):374–84. 10.1093/bfgp/elq020 20829207 PMC3080772

[66] Court F, Camprubi C, Garcia CV, Guillaumet-Adkins A, Sparago A, Seruggia D, et al The PEG13-DMR and brain-specific enhancers dictate imprinted expression within the 8q24 intellectual disability risk locus. Epigenetics Chromatin. 2014;7(1):5 10.1186/1756-8935-7-5 24667089 PMC3986935

[67] Venkatraman A, He XC, Thorvaldsen JL, Sugimura R, Perry JM, Tao F, et al Maternal imprinting at the H19-Igf2 locus maintains adult haematopoietic stem cell quiescence. Nature. 2013;500(7462):345–9. 10.1038/nature12303 23863936 PMC3896866

[68] Wood DL, Nones K, Steptoe A, Christ A, Harliwong I, Newell F, et al Recommendations for Accurate Resolution of Gene and Isoform Allele-Specific Expression in RNA-Seq Data. PLoS One. 2015;10(5):e0126911 10.1371/journal.pone.0126911 25965996 PMC4428808

[69] Blagitko N, Mergenthaler S, Schulz U, Wollmann HA, Craigen W, Eggermann T, et al Human GRB10 is imprinted and expressed from the paternal and maternal allele in a highly tissue- and isoform-specific fashion. Hum Mol Genet. 2000;9(11):1587–95. . 10861285 10.1093/hmg/9.11.1587

[70] Vilardi F, Lorenz H, Dobberstein B. WRB is the receptor for TRC40/Asna1-mediated insertion of tail-anchored proteins into the ER membrane. J Cell Sci. 2011;124(Pt 8):1301–7. 10.1242/jcs.084277 21444755 PMC3115773

[71] Petryszak R, Burdett T, Fiorelli B, Fonseca NA, Gonzalez-Porta M, Hastings E, et al Expression Atlas update—a database of gene and transcript expression from microarray- and sequencing-based functional genomics experiments. Nucleic Acids Res. 2014;42(Database issue):D926–32. 10.1093/nar/gkt1270 24304889 PMC3964963

[72] Uhlen M, Fagerberg L, Hallstrom BM, Lindskog C, Oksvold P, Mardinoglu A, et al Proteomics. Tissue-based map of the human proteome. Science. 2015;347(6220):1260419 10.1126/science.1260419 . 25613900

[73] Slabicki M, Theis M, Krastev DB, Samsonov S, Mundwiller E, Junqueira M, et al A genome-scale DNA repair RNAi screen identifies SPG48 as a novel gene associated with hereditary spastic paraplegia. PLoS Biol. 2010;8(6):e1000408 10.1371/journal.pbio.1000408 20613862 PMC2893954

[74] Ait Yahya-Graison E, Aubert J, Dauphinot L, Rivals I, Prieur M, Golfier G, et al Classification of human chromosome 21 gene-expression variations in Down syndrome: impact on disease phenotypes. Am J Hum Genet. 2007;81(3):475–91. 10.1086/520000 17701894 PMC1950826

[75] Rodriguez JM, Maietta P, Ezkurdia I, Pietrelli A, Wesselink JJ, Lopez G, et al APPRIS: annotation of principal and alternative splice isoforms. Nucleic Acids Res. 2013;41(Database issue):D110–7. 10.1093/nar/gks1058 23161672 PMC3531113

[76] Cunningham F, Amode MR, Barrell D, Beal K, Billis K, Brent S, et al Ensembl 2015. Nucleic Acids Res. 2015;43(Database issue):D662–9. 10.1093/nar/gku1010 25352552 PMC4383879

