# Supplementary figures and images for: Trisomy 21 Alters DNA Methylation in Parent-of-Origin-Dependent and -Independent Manners
Source: PLoS One. 2016 Apr 21;11(4):e0154108. doi: 10.1371/journal.pone.0154108 (PMC4839675; doi:10.1371/journal.pone.0154108)

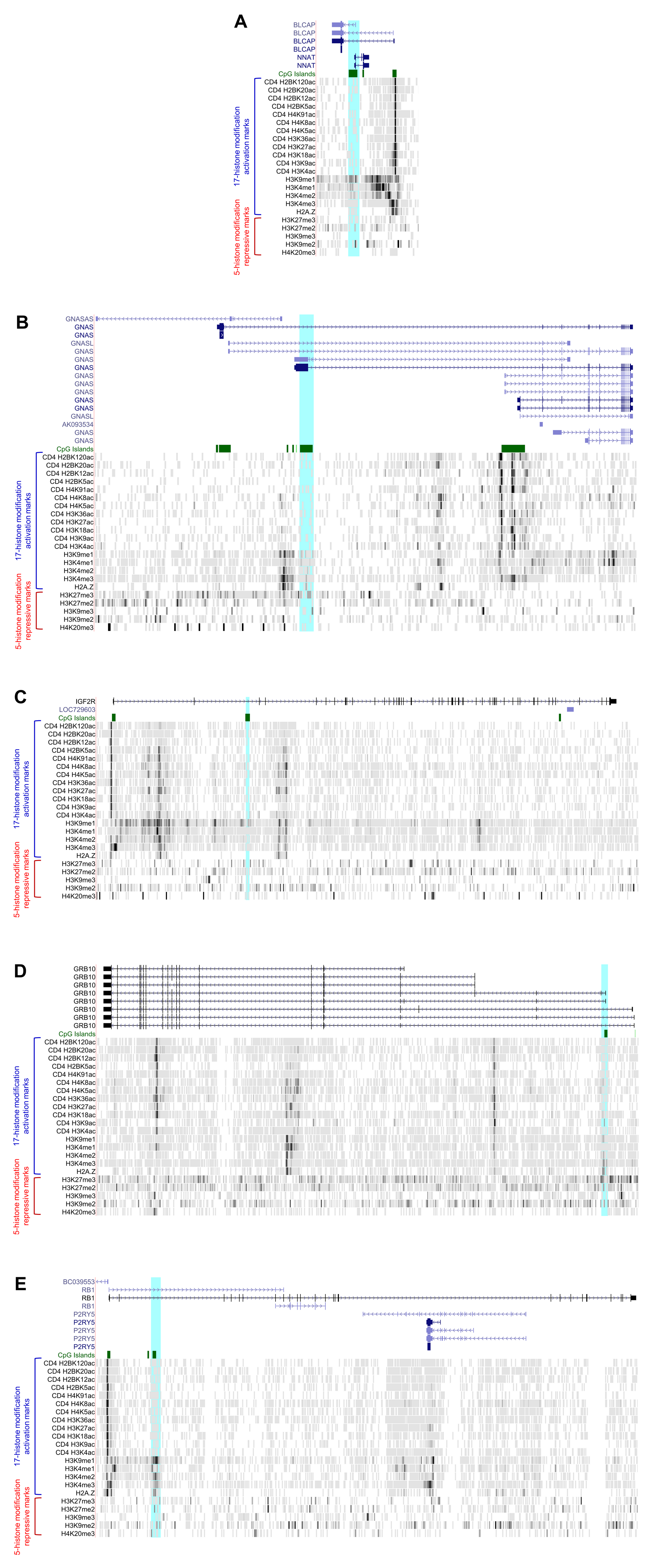

Supplement: S1 Fig — Graphical representation of the confluence of activating and repressive epigenetics histone modification marks for the known imprinted genes (A) BLCAP, (B) GNAS, (C) IGF2R, (D) GRB10 and (E) RB1. Shown are the 17-histone modification activation backbone module, and the 5-histone modification repressive module found in human CD4+ T cells [54]. Highlighted in light blue is the DMR in each gene. Composite of screenshots of the dataset viewed at the UCSC Genome Browser hg18 (http://genome.ucsc.edu). (TIF) [file pone.0154108.s001.tif]
